# Flexible Integration of Structurally Diverse Spatial Multi‐Omics With AgaeSMO

**DOI:** 10.1002/advs.78141

**Published:** 2026-10-12

**Authors:** Kunpeng Liu, Jinpo Su, Haotian Zhou, Yilin Shen, Mengke Qin, Yingjie Luo, Tiancai Wang, Zhenglin Chen, Yuanyuan Wang, Xiaokai Ma

**Affiliations:** ^1^ Center for Genomics and Biotechnology Fujian Provincial Key Laboratory of Haixia Plant Systems Biology Haixia Institute of Science and Technology College of Life Sciences Fujian Agriculture and Forestry University Fuzhou China; ^2^ Key Laboratory of Orchid Conservation and Utilization of National Forestry and Grassland Administration Fujian Agriculture and Forestry University Fuzhou China

**Keywords:** cross‐omics integration, graph attention network, morphomics integration, spatial multi‐omics, unpaired omics integration

## Abstract

Spatial multi‐omics (transcriptome, epigenome, proteome, metabolome, morphomics), provide near‐panoramic omics profiles across tissues. However, cross‐integration of structurally unpaired and underutilized morphomic datasets (e.g., Hematoxylin and eosin (H&E)‐stained images), remains a bottleneck due to inherent differences in spot structures, feature dimensions, and cellular heterogeneities. Here, we present AgaeSMO, an adaptive graph auto‐encoder for versatile cross‐modality integration of both structurally paired and unpaired spatial multi‐omics data. AgaeSMO encodes each omics modality from a cell type‐aware KNN (k‐nearest neighbor) feature graph and a spatial adjacency graph, while leverages GATs (Graph Attention Networks) as the backbone to fuse modalities by learning joint representations through an attention‐weighted module. It specially fuses structurally unpaired modalities, which applies a morphological transformation strategy by designing a cross‐guidance alignment among modalities through graph cross‐attention learning. H&E images are treated as a standalone morphomic modality (rather than auxiliary features) is specially fused with other modalities through image patch embeddings to fully utilize cellular morphological heterogeneities. AgaeSMO is a powerful tool can effectively integrate diverse omics modalities with both paired and unpaired spot architectures. These unique capabilities enable precise spatial domain delineation, micro‐environmental heterogeneity detection, compatible across broad platforms, that outperforming pre‐existing tools. AgaeSMO offers a versatile, robust framework for integrative spatial multi‐omics analysis and facilitates multimodal, biologically interpretable mining.

## Graphical abstract

AgaeSMO is a versatile and robust deep learning framework for flexible cross‐integration of spatial multi‐omics data. Leveraging a GATs network with a graph attention module, it learns joint embedding across diverse modalities, enabling integration of both structurally paired (e.g., spatial transcriptome‐epigenome, transcriptome‐proteome, transcriptome‐morphomics) and unpaired (e.g., spatial transcriptome‐metabolome) data. AgaeSMO delivers superior performance on multimodal downstream tasks while maintaining excellent biological interpretability.

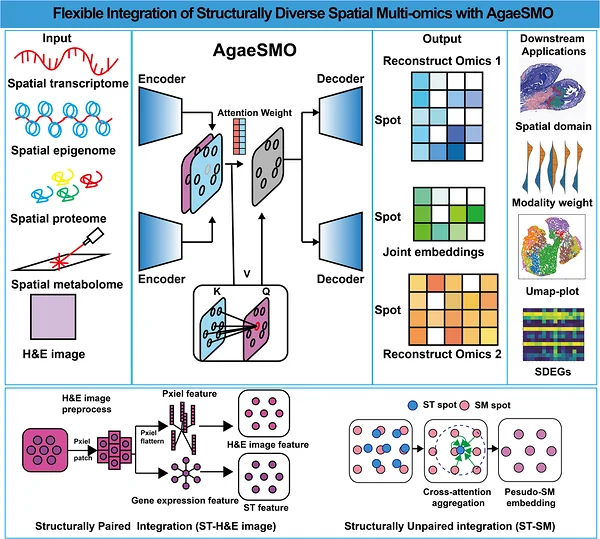

## 1 Introduction

The spatial multi‐omics technique on the same or adjacent slice aims to simultaneously capture information flowing on the central dogma, including ATAC (Assay for Transposase‐Accessible Chromatin), RNA, protein, and metabolites, as well as cell morphomic images. It brings multi‐perspective information to identify organizational heterogeneity, compensating for information that cannot be captured by single‐omics information, which may be necessary for identifying organizational heterogeneity [1]. Currently, there are techniques for single slice with dual‐omics, including spatial‐CITE‐seq [2], DBIT‐seq [3], Stereo‐CITE‐seq [4], spatial epigenome‐transcriptome co‐profiling [5], spatial multimodal analysis (SMA) protocol [6]. Moreover, the H&E (Hematoxylin and eosin) stained images provide much information in heterogeneity of cell morphologies in spatial omics. For instance, iStar [7] achieves super‐resolution of spatial transcriptomes based on H&E images. iSCALE [8] trained H&E images to predict gene expression by imaging‐based ST (Spatial Transcriptomics) platforms. They demonstrated that H&E images contain certain cellular information, thus achieving the conversion from H&E images to the transcriptome. However, H&E images has not been served as a modality for integration in spatial multi‐omics.

Even though the effective integration of multi‐modal spatial‐omics data still presents a significant challenge, arising from inherent differences in feature dimensions, data distributions, and the underlying spatial architectures of detection spots among modalities (e.g., 10x Visium for six‐neighborhoods and MALDI‐MSI (Matrix‐Assisted Laser Desorption/Ionization‐Mass Spectrometry Imaging) for four‐neighborhoods). Currently, there are tools designed for integrating multi‐omics acquired from the same slice, such as SpatialGlue [9], SpaMV [10], 3D‐OT [11], soFusion [12], COSMOS [13], TotalVI [14], MultiVI [15], MOFA+ [16]. SpatialGlue can integrate spatial multi‐omics with the same spot structure by Graph Convolutional Networks (GCN) [17] and attention aggregation. However, previous studies have shown that Graph Attention Neural Networks (GATs) [18] are more suitable for spatial domain recognition, such as STAGATE [19], which is based on GATs [18], and STMGraph [20], which is based on GraphMAE [21]. TotalVI [14], MultiVI [15], and MOFA+ [16] were designed for single‐cell multi‐omics, but they lose spatial information and may not be suitable for spatial multi‐omics. Currently, there is few tools designed to integrate two modalities that have different detection spot structures. For instance, the spatial positions of spots from 10x Visium and MALDI—MSI in the spatial multimodal analysis (SMA) protocol [6] do not correspond one‐to‐one, which consequently leads to the inability to establish a one‐to‐one correspondence between sample points. H&E‐stained images, as a type of pathological omics, can serve as diagnostic evidence for certain diseases [22]. However, current methods do not fully exploit H&E‐stained images. For instance, SpaGCN [23] uses the average RGB values of the surrounding spots as one basis for constructing the adjacency matrix. Although this approach considers the similarity of tissues in H&E staining images, it loses most of the features. H&E staining images have the potential to be served as a modality integrated with spatial multi‐omics.

To address the above limitations and challenges, we present AgaeSMO, an Adaptive Graph Attention Auto‐encoder for Spatial Multi‐Omics Integration. AgaeSMO encodes modal information using a spatial adjacency graph and KNN feature graph, subsequently applies GATs (Graph Attention Networks) as the backbone to extract modality features, which has more advantages than the GCN module in pre‐existing tools. It applies a cross‐attention mechanism to perform morphological transformation on unpaired structures of spatial multi‐omics data to solve the problem that pre‐existing tools cannot handle. It then uses an attention‐weight module to determine the modality weights according to their features, and aggregates them to obtain a joint representation as the input for unsupervised domain clustering. Besides, it constructs the H&E image features around the spots of transcriptomics as the embeddings for fusion to address the issue of integration of H&E‐stained images. Finally, through benchmarking tests, we assessed its performance in spatial domain delineation across multiple spatial‐omics platforms, and its role in enabling interpretable downstream biological analysis.

## 2 Results

### 2.1 Overview of AgaeSMO Model

AgaeSMO is a novel graph neural network that combines spatial and feature graphs for cross‐modality integration of spatial multi‐omics data (e.g., spatial‐transcriptome, ‐epigenome, ‐proteome, ‐metabolome, and morphomic) on the same slice (Figure 1a). For each spatial omics data, a spatial adjacency matrix and a cell‐aware adjacency matrix derived by KNN was separately constructed as the input of the encoder. The two modalities were adaptively re‐embedded, and the dimensionality of the node features was reduced through the GATs (Graph Attention Neural Networks) encoder, respectively (Figure 1b). The GATs were used as the backbone network, which enabled each spot to adaptively perform message passing with adjacent spots. In order to integrate the modalities of the spatial morphomic (e.g., H&E‐stained images) and spatial transcriptome, we segmented the slide image centered on the spatial location of each sequenced spot, and flatten the image patches into image embeddings. All the features within a certain spatial range of the spots were used as the morphomic modality for integrating with other modalities (Figure 1c). For the structurally unpaired spatial‐omics modalities, we implemented cross‐guidance alignments through cross‐attention mechanism (Figure 1d). In this approach, modality 1 (servers as Query, e.g. spatial transcriptome) computes the attention weights over adjacent regions of modality 2 (servers as Key, e.g. spatial metabolome). These weights were subsequently used to aggregate modality 2 features, thereby constructing the representations (servers as Value) of modality 2 corresponding to each spatial coordinate of modality 1. Through this process, the metabolomic signals around each spatial transcriptomic spot were queried by the cross‐attention mechanism, while the appropriate metabolomic representations associated with a given transcriptomic pattern were learned. To integrate the structurally paired modalities, modality‐aligned fusion was performed directly without the need for morphological transformation. During multimodal integration, attention weights were computed based on the respective features through an attention‐weight module, which determine weights of each modality based on their low‐dimensional representations. The attention weights were then used to fuse the modalities into the joint representations, which was subsequently applied to unsupervised domain clustering tasks (Figure 1b,e). The joint representations were decoded by the GATs decoders for feature reconstruction. Finally, the reconstructed features will be used to calculate the reconstruction loss with the original features, and then was passed through the encoder again to calculate the correspondence loss with the modality representations (Figure 1b). The joint representations obtained after AgaeSMO processing were used for unsupervised clustering to obtain the spatial domain, which is then applied to downstream mining tasks (Figure 1e).

**FIGURE 1:**
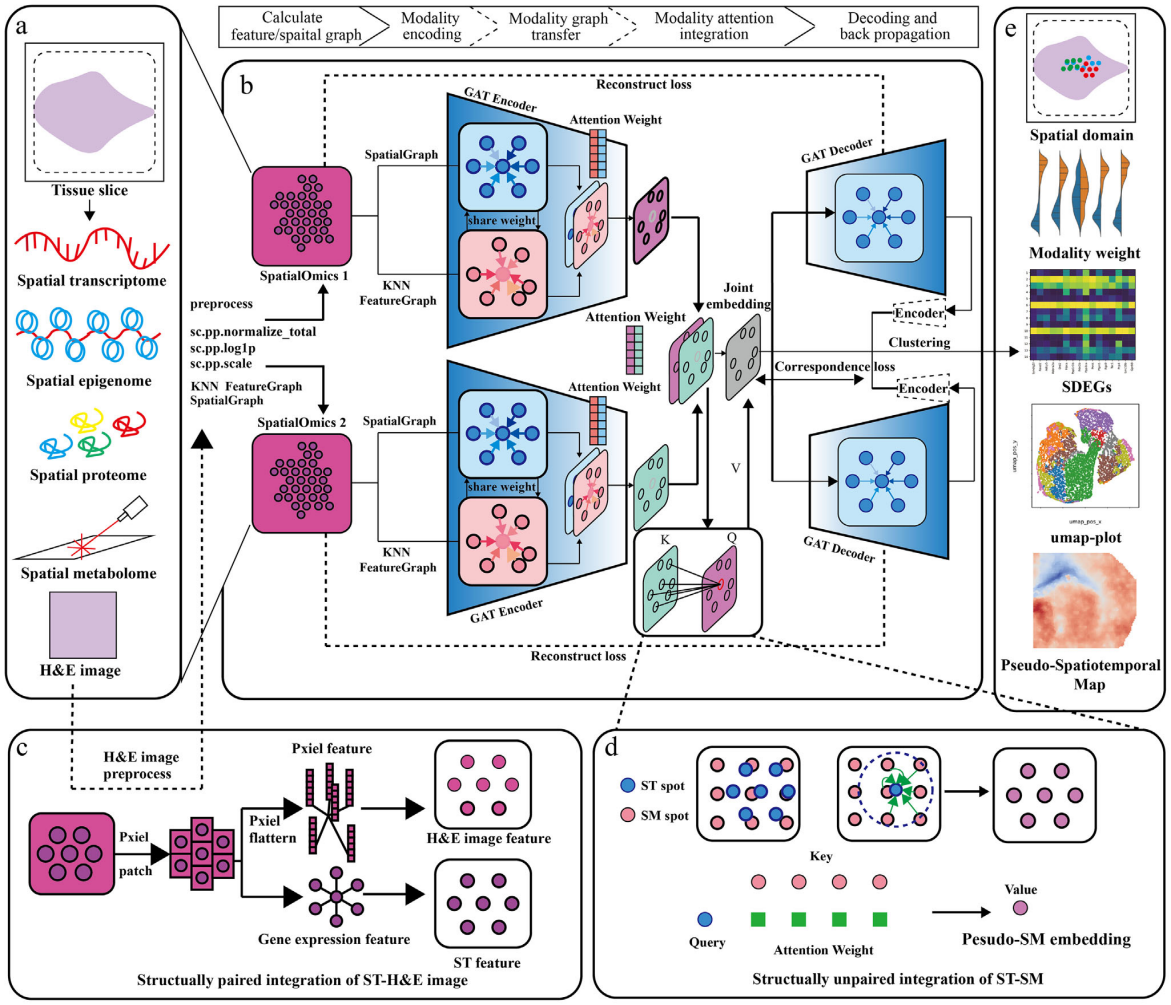
Overview of AgaeSMO. a Spatial multi‐omics data on the slice captures panoramic omics information of biological tissue. b AgaeSMO separately constructs a spatial adjacency graph based on a spatial adjacency matrix and a KNN feature adjacency graph based on a cell‐aware feature adjacency matrix. Two modalities are then encoded by a GATs encoder to generate low‐dimensional representations of each modality. Next, an attention module, which determines attention weights based on the low‐dimensional representations are employed to fuse the modal features for deriving a joint representation. Subsequently, the joint representation is decoded via the dual‐GATs decoder to accomplish feature reconstruction and calculate the reconstruction loss. Finally, the reconstructed features are re‐encoded by the encoder to compute the correspondence loss. Finally, the low‐dimensional joint representations are used for unsupervised clustering to obtain spatial domains. c AgaeSMO segments the H&E image centered on the RNA‐sequencing spots and flattens the image patches into image embeddings, then integrating spatial transcriptome and pathomic images. d AgaeSMO applies cross‐guidance alignments of modalities through a cross‐attention mechanism to process structurally unpaired spatial multi‐omics data. The modality 1 (servers as Query, e.g. spatial transcriptome) computes the attention weights over adjacent regions of modality 2 (servers as Key, e.g. spatial metabolome). These weights were subsequently used to transform the spot morphologies and aggregate features of modality 2 into that of modality 1, thereby constructing the representations (servers as Value) of modality 2 corresponding to each spatial coordinate of modality 1. e The low‐dimensional joint representations obtained after AgaeSMO processing are used for unsupervised clustering to obtain the spatial domain, which is then applied to downstream analysis, such as SDEGs (spatial differentially expressed genes), umap‐plot, and Pseudo‐Spatiotemporal Map.

### 2.2 Best Performance of AgaeSMO Than State‐of‐the‐Art Tools on Simulated Data and Spatial Multi‐Omics Datasets

We benchmarked the AgaeSMO model against current methods using simulated data and real datasets. For datasets with Ground Truth, we selected seven metrics, namely homogeneity, mutual information, v‐measure, AMI (adjusted mutual information), NMI (normalized mutual information), FMS (Fowlkes Mallows Score) and ARI (adjusted rand index) for evaluation. We tested eight competing methods: SpatialGlue [9], COSMOS [13], SpaMV [10], 3d‐OT [11], soFusion [12], TotalVI [14], MultiVI [15], MOFA+ [16] for multi‐omics integration and STAGATE [19], DeepST [24], SpaGCN [23], STMGraph [20] for spatial transcriptome modality.

We evaluated the performance of spatial domain clustering in AgaeSMO on simulated data (Table S1) consisting of two modalities (transcriptome and proteome) with complementary information of the ground truth (Figure 2a,b). AgaeSMO can effectively identify the spatial domains on five simulated datasets (Figure 2b; the results of the other four simulated datasets shown in Figures S1–S5). The clustering performance of AgaeSMO on the five datasets using seven metrics showed that AgaeSMO exhibited smaller variance compared to other schemes, which indicates its robust performance on simulated datasets (Figure 2c).

**FIGURE 2:**
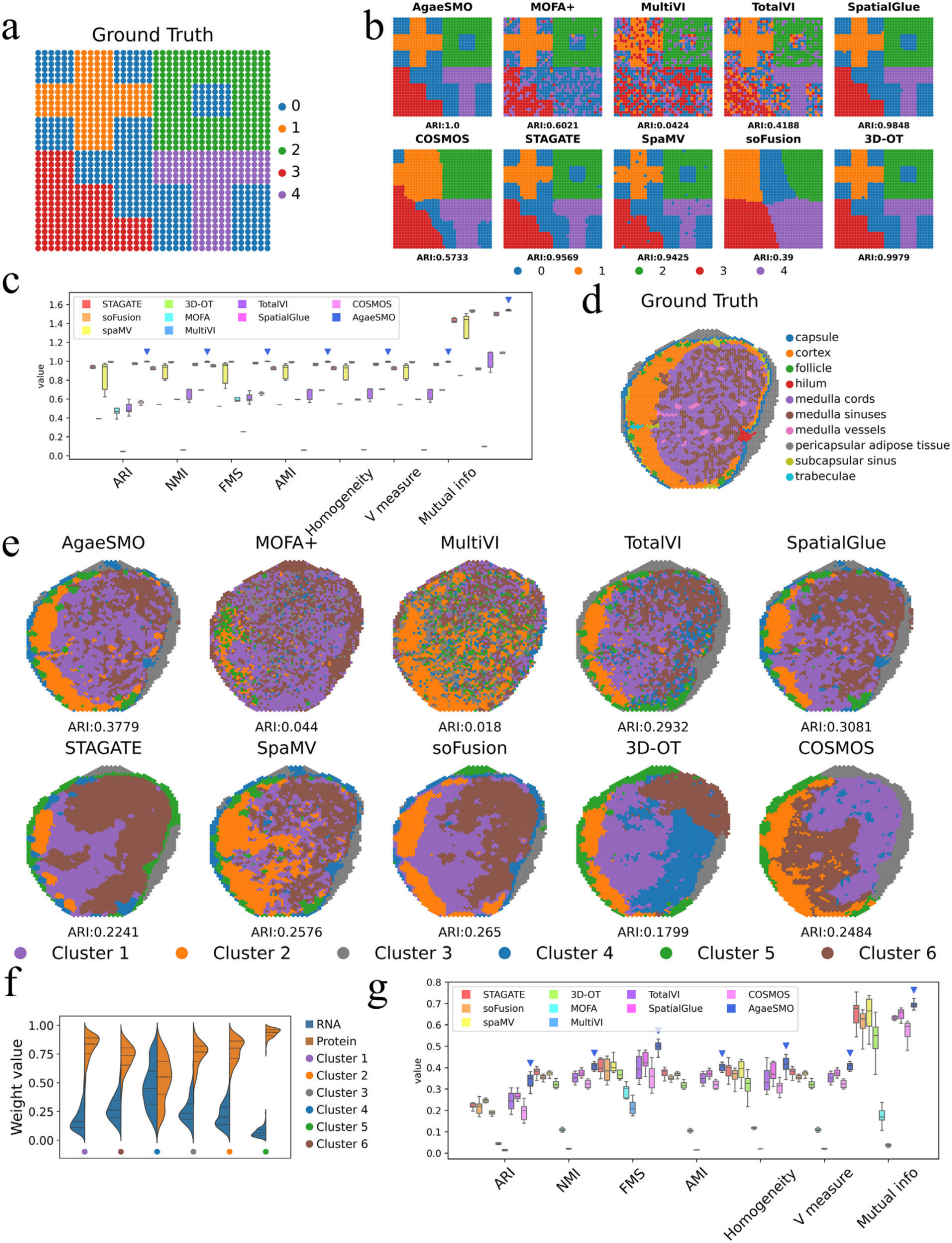
Benchmarking tests of AgaeSMO against existing methods on simulated spatial multi‐omics datasets and the spatial transcriptome‐proteome dataset of Human Lymph Node A1. a Ground truth of spatial multi‐omics simulation datasets (transcriptome and proteome). b AgaeSMO outperforms existing methods for deciphering spatial domains in simulation datasets of spatial transcriptome and spatial proteome. c Comparison of clustering metrics among AgaeSMO and other algorithms in five simulation datasets (*n* = 5 simulated samples). d Ground truth of Human Lymph Node A1 datasets. e Results of deciphering the spatial domain in Human Lymph Node A1 datasets using AgaeSMO and other methods. f Modality weights for of Human Lymph Node A1 datasets using AgaeSMO cluster results. g Comparison of clustering metrics among AgaeSMO and other algorithms from eight resolution classes (4 to 11) of Human Lymph Node A1 datasets.

Benchmarking tests for AgaeSMO against eleven methods were also performed on in‐house human lymph node datasets of section A1 with ground truth (Figure 2d and Table S1) generated using 10x Genomics Visium RNA and protein co‐profiling technology. The structure of the ground truth included the outer layers of the bulb comprised the pericapsular adipose tissue and capsule, while the core internal structures consisted of the cortex and medulla (sinuses, cords, and vessels) (Figure 2d). AgaeSMO outperformed all other methods with an ARI of 0.3779. All of the methods could identify the paracortex area (T cell zone, Figure 2e, cluster 2), however, the performance of MOFA+ and MultiVI was not well. MOFA+ could recognize areas smaller than the ground truth of the paracortex domain, while MultiVI exceeded this domain. AgaeSMO, TotalVI, SpaMV and Spatial Glue were able to identify the follicle area, while MOFA+, MultiVI, and all the single‐omic models were unable to recognize this region. Specifically, AgaeSMO was able to recognize the spatially dispersed distribution characteristics of medulla sinsuses regions, which could not be achieved by single‐omics algorithms and SpatialGlue, soFusion, 3d‐OT (Figure 2e, cluster 6; Figure 2d, medulla sinsuses region, Figure S6a). Even though the existing methods employed the scheme to make the clustering edges smoother, these approaches were not suitable to distinguish the medulla sinuses region (Figure 2e cluster 6, Figure 2d medulla sinuses). The modality weights were different for each cluster. The cluster 1, 2, 3, 5, and 6 was predominantly performed by the proteome modality, while in cluster 4 (hilum), the weights of RNA and proteins were nearly equivalent (Figure 2f,d). We further evaluated the model performance across a range of cluster numbers (6 to 11) by calculating the supervision metrics separately against the ground truth. The median score of AgaeSMO outperforms other methods in all the metrics (Figure 2g, AgaeSMO ARI = 0.3499, NMI = 0.4089, FMS = 0.4998, AMI = 0.4053, HMG (Homogeneity Score) = 0.4160, VMS (V‐measure Score) = 0.4089, MIS (Mutual Infomation Score) = 0.6930). Then, we conducted ablation experiments on human lymph node A1 dataset to validate the feature extraction and integration capabilities of each module in AgaeSMO. We compared the AgaeSMO full model against two variants: 1) GATs module replaced by GCN encoder‐decoder (GCN En/Decoder), and 2) feature aggregation module substituted by simple concatenation (e.g. Concatenation) (Figure S7). The absence of GATs introduces substantially more noise in clustering. The GCN En/Decoder (ARI = 0.2942) failed to identify the capsule region, and misclassified a region within the medulla cords. While the direct Concatenation (ARI = 0.3192) could identify the capsule region, but still misidentified the domain within the medulla cords.

Next, we applied AgaeSMO to analyze an experimental dataset containing P22 mouse brain coronal sections (Table S1) with a joint profiling of spatial chromatin accessibility (ATAC‐seq) and spatial transcriptome (RNA‐seq) [5] (Figure 3a). We first applied SpaGCN, STAGATE, and STMGraph to the spatial transcriptome modality and then applied COSMOS, SpaMV, 3d‐OT, soFusion, MultiVI, SpatialGlue, MOFA+, and AgaeSMO to integrate the datasets with two omics. AgaeSMO achieved the highest ARI value of 0.3908 in deciphering the spatial domain (Figure 3b). COSMOS, MultiVI, SpatialGlue, SpaGCN were only able to identify ccg (genu of corpus callosum) (Figure 3a, ccg/aco; Figure S6b), while AgaeSMO, SpaMV, 3d‐OT, and SpatialGlue were able to deciphering entire CP (caudoputamen) (Figure 3b). Furthermore, we evaluated the performance of AgaeSMO with other methods based on multiple indicators across cluster numbers ranging from 4 to 11. AgaeSMO outperformed other methods in terms of the median of ARI and FMS values (Figure 3c). Next, we generated UMAP visualization and pSM (pseudo‐Spatiotemporal Map) from the low‐dimensional representation by SpatialGlue, COSMOS, and AgaeSMO (Figure 3d,e). Based on the pSM inferred from the AgaeSMO representation, we calculated the Pearson's correlation between each gene and the pSM value. The top three and bottom three genes of the correlation coefficient were regionally expressed in the ccg/aco (genu of corpus callosum/anterior commissure olfactory limb), CP (caudoputamen), and L1‐L3 regions (Figure 3f). *X3110035E14Rik*, *Mbp*, and *Mobp* were negatively correlated with pSM, while *Meg3*, *Gm26924*, and *Dgkb* were positively correlated with pSM (Figure 3f). The *Mbp* encoding the myelin basic protein (MBP) was revealed as a marker for the myelination process in the rat brain during development from postnatal day 1 to day 21 [25], while *Mobp* encoded myelin protein [26].

**FIGURE 3:**
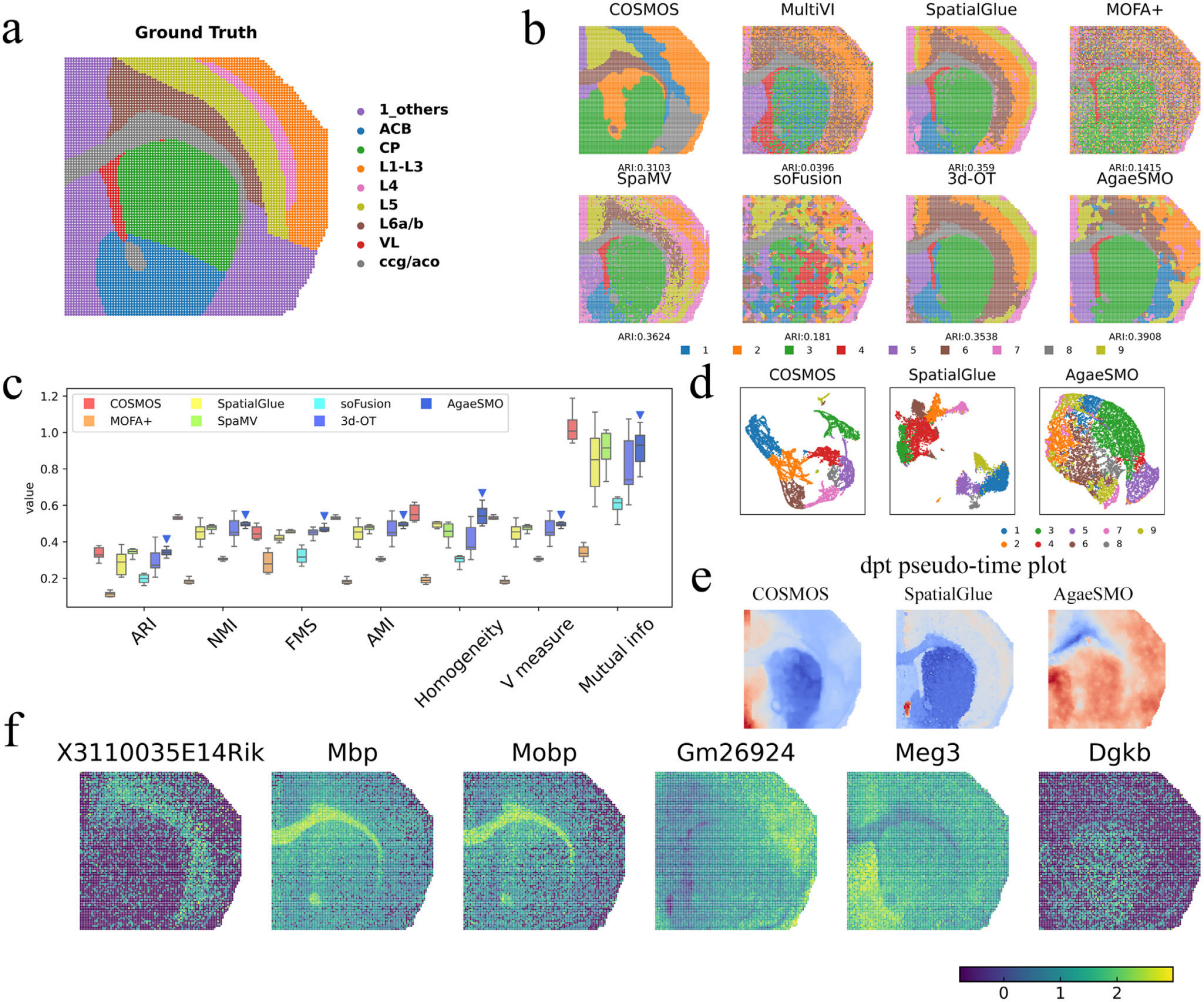
Benchmarking tests of AgaeSMO against other methods on integrating the spatial epigenome‐transcriptome dataset. a Ground Truth of the ATAC‐RNA‐seq Dataset of a P22 Mouse Brain coronal section. b Comparison of clustering results of AgaeSMO and other methods. AgaeSMO outperforms other methods with 0.3908 of ARI. c Comparison of clustering metrics among AgaeSMO and other algorithms from eight resolutions classes (4 to 11) of the ATAC‐RNA‐seq Dataset. d UMAP plot of P22 Mouse Brain coronal section datasets after clustering by AgaeSMO, COSMOS, and SpatialGlue. e Pseudo‐spatiotemporal maps (pSM) generated by AgaeSMO, SpatialGlue, and COSMOS. f Spatial mapping of gene expressions for brain development genes that negatively (*X3110035E14Rik*, *Mbp*, *Mobp*) and positively (*Meg3*, *Gm26924*, *Dgkb*) correlated with pSM, identified by AgaeSMO.

### 2.3 AgaeSMO Integrates Spatial Modalities With Different Spots Structures

The performance of AgaeSMO on the SMA datasets (Table S1) was compared with the algorithms of spatial domain clustering in single‐omics. The clustering algorithms, like SpatialGlue [9], COSMOS [13] were not included in the comparisons because they were specially designed for RNA‐protein and RNA‐ATAC‐seq integration. Moreover, existing multi‐omics, such as SpatialGlue [9], COSMOS [13], SpaMV [10], soFusion [12], and 3d‐OT [11] integrated recognition spatial domain algorithms did not have the ability to integrate different spot structures or simply average the MS signals near the ST spot locations as model input, such as SpaMV, because the model requires tensors with the same number of spots for attention aggregation, which relies on detecting multi‐omics in the same spot (Figure S8). Because of different structures between 10x Visium (six neighborhoods) and MALDI‐MSI (four neighborhoods) data, in AgaeSMO, a cross‐attention module for different spot structures was designed by calculating the attention of a single spot in the RNA modality and its k adjacent MSI (Mass Spectrometry Imaging) spots. It summed the attention‐weighted adjacent MSI spots to generate new sampling points at this RNA spot location while retaining MSI information, providing a tensor with the same structure as the spatial RNA‐seq spots for the next step of attention integration (Figure 1d).

In the SMA datasets, three mouse brain coronal section slices (Table S1) were obtained from mice treated with 6‐OHDA, which induced a mouse model of PD (Parkinson's disease), we annotated the intact and lesioned parts based on the annotations of the original data (Figures 4a and S9a, Ground truth). The left and right CP (Caudoputamen) were different, with the left part (Figure 4a–c, red circle) being intact and the other right part (Figure 4a–c, blue circle) being lesioned. There are differences in metabolite intensity in the left and right CP (Caudoputamen) regions, such as dopamine (Figure 4b), which is related to PD (Parkinson's disease) [6]. Theoretically, the clustering in the left and right CP (Caudoputamen) regions (red and blue circles) should be different (Figure 4c). When using the single RNA modality, AgaeSMO clustered the two CP (Caudoputamen) regions as the same domain (Figure 4c AgaeSMO RNA), while single MSI modality clustering showed slight differences (Figure 4c AgaeSMO MSI). However, after integration of RNA‐MSI modalities, two CP (Caudoputamen) regions were clustered separately well as two domains (Figure 4c AgaeSMO). AgaeSMO could convert MSI information and integrate it with RNA modality to distinguish different CP (Caudoputamen) regions. Compared with the spatial domain recognition of other single‐omics algorithms, only STAGATE could recognize different CP (Caudoputamen) regions in three slices, while STMGraph can recognize one (Figure S9a). However, on the SMA 109 B1 dataset, AgaeSMO can distinguish ACB‐intact with ACB‐lesioned regions, whereas STAGATE and STMGraph cannot (Figure S9a,b red arrow). The two well‐separated CP clusters (clusters 2, 6 &10) generated by AgaeSMO, were basically contributed by attention weights of the spatial transcriptomics modality but strongly facilitated by the spatial metabolomics modality (Figures 4c,d and S10a).

**FIGURE 4:**
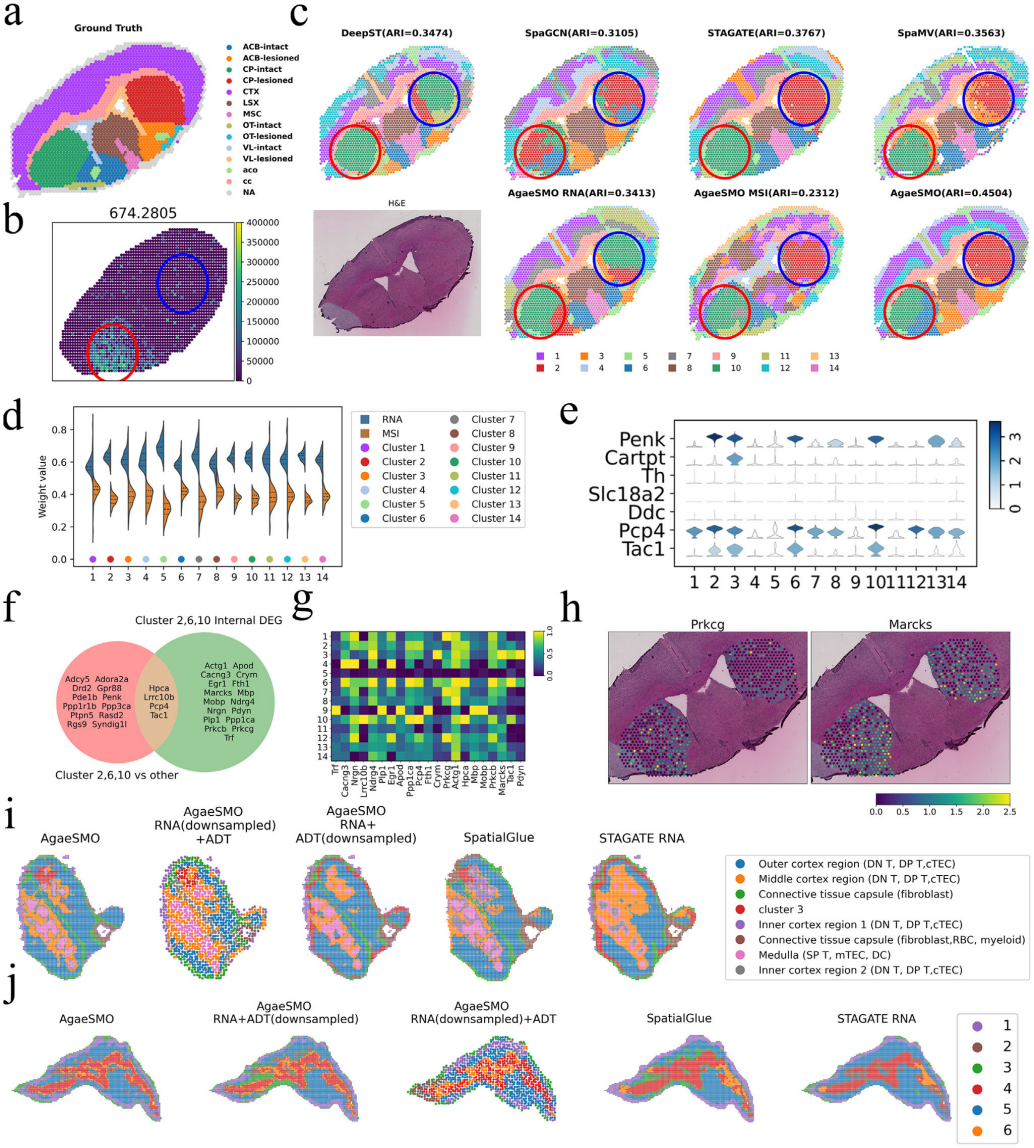
AgaeSMO integrates spatial‐omics data of different spot structures. a The Ground Truth of SMA 109 A1 datasets. b Dopamine (m/z 674.28) ionic intensity in whole slice, in the two CP (Caudoputamen) regions with intact (left red circle) and lesion (right blue circle), with intact CP shows a higher intensity of dopamine level. c Comparison of clustering abilities between AgaeSMO and other methods. “AgaeSMO” means the clustering result after two modalities integration, while “AgaeSMO‐RNA” and “AgaeSMO‐MSI” are the clustering results of the latent variables of mRNA and MSI modality, respectively. d Modality weight of each cluster. The modality weight is dominated by the mRNA modality. e Expression patterns of PD (Parkinson's disease) related genes in each cluster. f Venn plots between the upregulated SDEGs between cluster 2,6,10 versus other clusters, and the internal SDEGs among cluster 2, 6 and10. The red part of the diagram represents the top ten upregulated SDEGs in clusters 2, 6, and 10 compared to other clusters, while the green part represents the top ten upregulated SDEGs of pairwise comparisons among clusters 2, 6, and 10. g Heatmap of SDEGs of cluster 2 versus 6 versus 10. h The spatial expression patterns of *Prkcg* and *Marcks* genes. i Comparison of clustering performance among AgaeSMO, SpatialGlue and STAGATE on complete and downsampled mouse thymus 1 datasets, and mouse thymus 2 datasets (j).

By downstream SDEGs (spatial differentially expressed genes) analysis according to clustering results, the specific/upregulated SDEGs of PD‐related genes in each cluster were determined (Figure 4e–h). *Th*, *Slc18a2*, *Ddc* are key gene of dopaminergic pathway [27], while *Pcp4*, *Tac1* are positively correlated with dopamine level, and *Penk*, *Cartpt* are negatively correlated with it [6]. As expected, *Penk* and *Pcp4* was highly expressed in clusters 2, 6, and 10 corresponding to the CP (Caudoputamen) region with high dopamine levels (Figure 4b, c). Among the SDEGs identified in each cluster and between clusters 2, 6, and 10 (Figure 4f: cluster 2, 6, 10 vs others; Figure S10b–d), *Prkcg* has been shown to cause Parkinson's syndrome when mutated in rat [28]. While, *Marcks* is a marker of neurite degeneration at the pre‐aggregation stage in PD/DLB pathology [29]. *Marcks* and *Prkcg* were DGEs detected internally by cluster 2, 6, and 10 (Figure 4f: cluster 2, 6, 10 internal SDEGs, 4h) rather than these cluster vs others (Figure 4f cluster 2, 6, 10 vs other, 4h), which indicates the necessity of detecting three differential regions within CP (Caudoputamen) (Figure 4a,c,e). To investigate the integration capability of each module in AgaeSMO for unpaired modalities, we further conducted ablation experiments on the SMA 109 B1 dataset via comparing AgaeSMO full model against three variants: 1) GATs module replaced by GCN encoder‐decoder (GCN En/Decoder), 2) cross‐modal alignment module without attention (e.g., Alignment w/o Attn), and 3) feature aggregation module substituted by simple concatenation (e.g., Concatenation) (Figure S11). The absence of GATs introduces substantially more noise in clustering performance. GCN En/Decoder (ARI = 0.2229) failed to identify both CP‐intact/lesioned regions and ACB‐intact/lesioned regions. Besides, the Alignment w/o Attn (ARI = 0.3423) and direct Concatenation (ARI = 0.3193), could not distinguish the ACB‐intact/lesioned regions in the SMA 109 B1 dataset.

Furthermore, we swapped the modality order to assess their impacts on clustering performance on both SMA 109 A1 and 038 B1 datasets. On the 109 A1 dataset, AgaeSMO could distinguish intact from lesioned regions (ARI = 0.450) (Figure S12a, AgaeSMO MSI to RNA). After modality swapping, AgaeSMO still distinguished these regions, but introduced a little noise compared with pre‐swapping (Figure S12b, AgaeSMO RNA to MSI). Due to inconsistent structure with ground truth after swapping, the ARI could not be computed. On the 038 B1 dataset, clustering performance kept relatively consistency regardless of modalities swapping, while AgaeSMO still maintained spatial structures and successfully distinguished CP‐intact/lesioned regions (Figure S12c,d, AgaeSMO). In both datasets, before and after swapping, RNA alone preserved accurate domains but failed to distinguish intact/lesioned regions, due to the absence of MSI information, while MSI alone remained noisy.

To validate the cross‐integration performance of AgaeSMO on unpaired high‐resolution spatial omics datasets, we utilized the Stereo‐CITE‐Seq (RNA+ADT) Mouse Thymus datasets with downsampled treatments (Figures 4i,j and S13; Table S1). We defined circle center and radius for transcriptomic and proteomic data, and averagely weighted original spots within radius based on UMI (Unique Molecular Identifier) counts. Random offsets uniformly sampled from −50 to 50 to *x*–*y* coordinates of each center (Figure S13a). The following dataset combinations were obtained for Mouse Thymus 1 and 2 datasets: 1) RNA+ADT, 2) RNA+ADT (downsampled), and 3) RNA (downsampled)+ADT. AgaeSMO was tested on these datasets comparing with STAGATE. In Mouse Thymus 1, STAGATE RNA failed to identify the connective tissue capsule (fibroblast) region, whereas STAGATE ADT achieved this (Figures 4i and S13b, red arrow). In contrast, AgaeSMO accurately identified this region by integrating paired/unpaired datasets in both original Mouse Thymus 1 and RNA+ADT (downsampled) datasets. In Mouse Thymus 2, spatial domain identification was robust to downsampling (Figures 4j and S13c). For example, clusters 1 and 6 exhibited spatial distributions similar to those of the complete dataset in both downsampled combinations.

### 2.4 AgaeSMO Integrates H&E Stained Image as a Morphomic Modality With Spatial Transcriptome

H&E stained images contain the heterogeneity information of cell morphologies of whole slide in both animals and plants (Figure 5a,b,g–i). AgaeSMO exhibited the abilities for integrating the H&E‐stained images as one formal morphomic modality for domain clustering of spatial multi‐omics. It first segmented the H&E image as the patches. Each patch was defined a square block centered around a RNA sequencing spot, and then flattened to generate a pixel embedding (Figure 1c; Figures S14 and S15). The RNA expression embeddings (modality 1) and pixel embeddings (modality 2) were subsequently served as the input modalities to the AgaeSMO.

**FIGURE 5:**
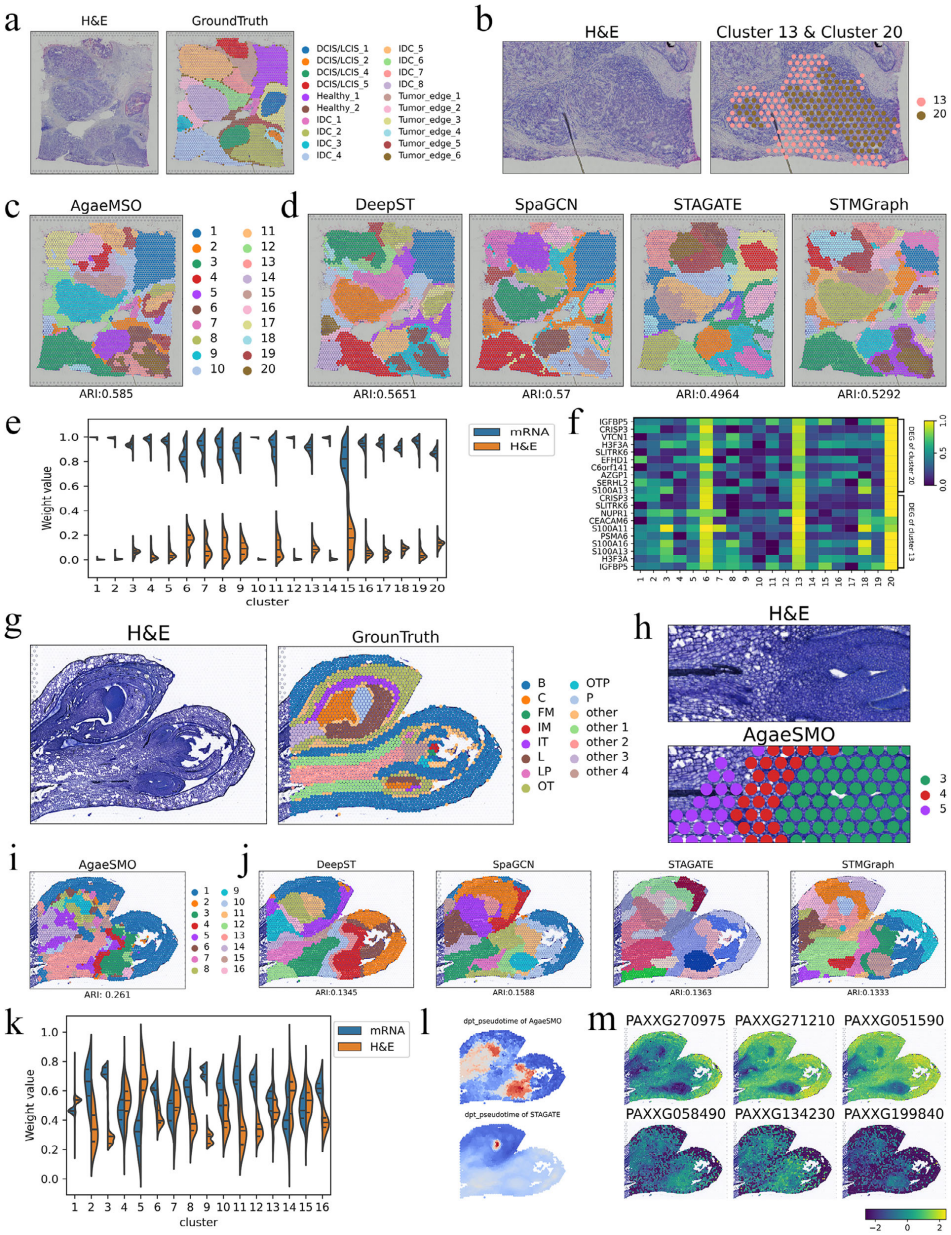
AgaeSMO integrates H&E images as a morphomic modality for spatial multi‐omics analysis. a Ground truth and H&E image of Human Breast Cancer datasets. b Different tissues exhibit distinct cellular morphologies. c Clustering performance of AgaeSMO on Human Breast Cancer datasets. d Clustering performance of existing methods on Human Breast Cancer datasets. e Modality weight of each cluster on Human Breast Cancer datasets, with clusters 20, 13 share more H&E image modality weight compare with other cluster. f Heatmap of the expression profiles of the top 10 spatially differentially expressed genes (SDEGs) from clusters 20 and 13 identified based on AgaeSMO clustering results across all clusters. g H&E image and manual annotation of spatial domain based on the H&E labeling of orchid flower bud slices 1. h Different tissues exhibit distinct cellular morphologies in orchid bud datasets. i Clustering performance of AgaeSMO on orchid bud datasets. j Clustering performance of existing methods on orchid bud datasets. k Modality weight of each cluster on orchid bud dataset, with cluster 4, 5 share more H&E image modality weight compare with other cluster. l Pseudo spatial temporal maps (pSM) generated by AgaeSMO consistent with the anatomical structure of the orchid flower bud, compare with that generated by STAGATE. m Spatial mapping of gene expressions of SDEGs that negatively (*PAXXG270975*, *AXXG271210*, *PAXXG051590*) and positively (*PAXXG058490*, *PAXXG134230*, *PAXXG199840*) correlated with pseudotime, as identified by AgaeSMO.

Compared to other solutions, AgaeSMO achieved the highest deciphering accuracy of clustering in the Human Breast Cancer dataset (Table S1), with an ARI value of 0.585, after integrating the modalities of H&E‐stained image with RNA sequencing (Figure 5c). Although the modality of RNA sequencing generally showed more weight in each cluster (Figure 5e), the modality of H&E image in clusters 6, 7, 8, 9, 13, 15, and 20 showed increased weight, especially in clusters 20 and 13, where the tissue morphologies showed differences in H&E staining (Figure 5b). Some candidate SDEGs (e.g. *IGFBP5, EFHD1, AZGP1*), were successfully detected in clusters 13 and 20 (Figures S16–S18 and Table S4) by AgaeSMO, which might be as the downstream targets (Figure 5f). For instance, the expression level of *IGFBP5* is associated with breast cancer susceptibility [30], while *EFHD1* promotes breast cancer progression through estrogen signaling [31], *AZGP1* could promote the proliferation, migration, and invasion ability of breast cancer cells [32].

We further applied AgaeSMO on the spatial data of the orchid *Phalaenopsis* flower bud. We manually annotated the spatial domain based on the H&E labeling of flower bud slices 1 (Figures 5g and S19; Table S1). AgaeSMO utilized more features of morphomic modality (H&E image) for orchid dataset than those of breast cancer dataset. Compared to other solutions, AgaeSMO performed well with an ARI value of 0.261 (Figure 5i), especially with relatively complete recognition of the Bract, Column and Rostellum, Pollinium regions (Figure 5i,j cluster 1, 8, 9). Moreover, in the Bract region, the weight of the H&E modality is greater than that of the RNA modality (Figure 5k, cluster 1). Clusters 4 and 5 exhibited more weights of the H&E modality compared to other clusters, which is also reflected in the cell morphology (Figure 5h,k). Next, we used the low‐dimensional representations generated by AgaeSMO to generate a pseudo‐spatiotemporal map (pSM) by running the diffusion pseudo‐time. Compare with STAGATE, pSM of AgaeSMO was consistent with the anatomical structure of the orchid flower (Figure 5l). Next, we calculated the Pearson correlation between pSM value and gene expression. The top three (*PAXXG058490*, *PAXXG134230*, *PAXXG199840*) and bottom three (*PAXXG270975*, *PAXXG271210*, *PAXXG051590*) genes were identified based on the correlation coefficient (Figure 5m). *PAXXG134230* encoding osmotin‐like protein involved in improving plant salt tolerance [33], In the in vitro tobacco thin cell layer explant system of tobacco, osmotin mRNA expression has been implicated in flowering developmental processes, as well as in the phenotypic outcomes of hormone treatments that induce floral transition [34]. *PAXXG199840* is *RIC10* gene which induce multi‐process regulation like ROP‐GTPase regulatory system, ROP GTPase effector activities and as GTPase effector (RIC). Previous studies confirm that RIC10 promotes pollen tube elongation [35].

## 3 Discussion

Our developed AgaeSMO addressed various challenges in the integration of spatial multi‐omics data. AgaeSMO leverages the GATs as the backbone networks to adaptively pass message between nodes. It is the first algorithm using a graph attention module to integrate modalities with different spot structures, and incorporates all features of H&E‐stained images as a morphomic modality for spatial multi‐omics integration. Its unique capabilities enable precise spatial domain delineation, micro‐environmental heterogeneity detection, compatible across broad platforms, that outperforming pre‐existing tools.

AgaeSMO adopts Graph Attention Networks (GATs) as the backbone architecture, achieving better feature extraction (Figure 1b). Unlike GCN [17], GATs introduces an attention mechanism that dynamically assigns distinct importance weights to neighboring nodes, enabling the model to selectively focus on relevant neighbors while suppressing noise from less informative neighbors [18]. This adaptive aggregation is particularly advantageous for multi‐omics integration, as it allows the network to learn context‐aware representations to capture more heterogeneous features [19]. GCN‐based tools like SpatialGlue [9] and COSMOS [13] have limited capabilities to achieve this goal, as their static aggregation weights preventing adaptive message passing, thus failed to perform effective feature extraction during integration [18]. Our ablation experiments further demonstrate that the GATs encoder‐decoder contributes most significantly to overall model performance, that outerperforming the GCN‐based clustering (Figures S7 and S11), confirming its effectiveness in learning robust, discriminative embeddings for spatial multi‐omics integration clustering.

AgaeSMO specially fused the structurally unpaired spatial multi‐omics by designing a cross‐guidance alignment among modalities through a graph cross‐attention mechanism. It uses modality 1 representations as queries to obtain corresponding modality 2 representations and aggregate them to the spatial positions of modality 1, realizing the adaptive re‐allocation of the spatial positions of modality 2. This strategy substantially solves the problem of integrating structurally unpaired modalities of spatial transcriptome and metabolome of SMA protocol data that SpatialGlue [9] and COSMOS [13] cannot handle (Figure 4). Nevertheless, translation‐based models provide an alternative for integration of unpaired modalities, which align modalities by predicting one at positions of another, and fuse them via integration tools for paired‐modalities. For example, SpatialEX+ [36] uses RNA information to infer metabolome, while NicheTrans [37] obtains cell niches from transcriptome and encodes them via Transformer to derive metabolome. Compared with direct modalities alignments via attention using AgaeSMO, translation models may require more parameters or computational resources, but they open up a new avenue for interpretability.

H&E‐stained images containing substantial morphological information (e.g. cell morphological micro‐environmental heterogeneities) that should be regarded as a morphomic modality on par with other modalities. Traditional spatial algorithms such as SpaGCN [23], STAGATE [19], DeepST [24], and STMGraph [20] neither considered or partially used H&E image information as auxiliary input for constructing a GNN graph. Our AgaeSMO integrates H&E images as a formal morphomic modality by extracting spot‐level cell morphological embeddings from resampled H&E images and fuse them with other modalities. It performed well when two omics hold distinct, complementary features and fully utilized the information of H&E images, even though the expanded image feature dimension (768) remains far below transcriptomic dimension (∼3000). Enhanced by H&E image modality, the spatial clustering shows superior performance than use spatial transcriptomics alone, yielding more biologically meaningful outcomes in downstream analyses (Figure 5). This integration may not be only limited to H&E images, but also for other morphomic modalities such as hyperspectral and confocal microscope images.

AgaeSMO shows superior abilities of cross‐integration of spatial‐omics modalities. We systematically benchmarked it on five simulated spatial datasets of transcriptome‐proteome, one spatial epigenome‐transcriptome, three spatial transcriptome‐metabolome, and two spatial transcriptome‐morphomic datasets. All the comparisons demonstrated that AgaeSMO outperformed other methods with the compatibilities of fusing structurally unpaired modalities and integrating the morphomic modality. Powered by cross‐modality attention for modality fusions, AgaeSMO enables discovering the micro‐environmental heterogeneity regions and key functional genes with biological interpretabilities. Notably, after integration of RNA‐MSI modalities of mouse brain coronal datasets, two CP (Caudoputamen) regions were clustered separately well as two domains by AgaeSMO rather than other methods (Figure 4c) or prior analyses [6]. Based on this micro‐environmental clustering, the critical Parkinson's disease‐related genes (*Prkcg, Marcks, Th, Ddc*) mediating dopaminergic dysfunction and neurite degeneration was successfully detected. When integrated spatial transcriptome with morphomic data (H&E images), AgaeSMO successfully detected the key genes (*IGFBP5*, *EFHD1*, *AZGP1*) driving breast cancer progression, as well as genes (*RIC10*, encoding osmotin‐like protein) regulating orchid flower bud development.

Overall, AgaeSMO provide a superior tool for spatial multi‐omics studies. With the development of spatial omics technology, we anticipate that AgaeSMO will reveal more possibilities, as the increasing amount of information‐rich spatial multi‐omics data. Although measuring multiple omics on the same tissue slice remains a challenge, AgaeSMO is, in principle, extensible to the integration of three or more modalities, pending experimental validation.

## 4 Method

### 4.1 Data Preprocessing

Omics data was preprocessed by Scanpy [38] package. For gene expression, we filtered out mitochondrial genes, then filtered out genes with expression in less than three cells, and selected the top highly variable genes then through normalize and log transfer. All steps were implemented through scanpy. Preprocessing of each dataset can be found in Table S2

### 4.2 Embedding of H&E staining images

Let expression matrix $A\in R^{n\times m}$, n represents spot number. And each spot centre location in H&E image (*x_i_*,*y_i_*), select four anchor points based on the center point (*x_i_*,*y_i_*) and use them to segment the image, pixel coordinates of the patch fellows:

(1) $$P_{i}=\left\{\left(x,y\right)|\left|x-x_{i}\right|<r,\left|y-y_{i}\right|<r\right\}$$

$P_{i}\in R^{2r\times 2r\times 3}$, each spot has a patch,r is a hyperparameter. Patch embedding $P\in R^{n\times 2r\times 2r\times 3}$, then flatten the last three dimensions fellows:

$$P\in R^{n\times 12r^{2}}$$

$A\in R^{n\times m}$ and $P\in R^{n\times 12r^{2}}$ represent the gene expression and H&E staining images embedding of each spot, respectively. *A* and *P* are transcriptomics and pathomics as inputs for AgaeSMO.

### 4.3 Construction of KNN Feature Graph

In order to capture the neighbor relationships of remote spots and transmit information to them through GATs. First, we performed PCA dimensionality reduction on the expression matrix of highly variable genes $A\in R^{n\times m}$ to $A\in R^{n\times k}$ (k dimensions), then calculated the KNN graph and specify k nearest nodes as neighboring nodes for each node. The KNN graph was constructed by scikit‐learn [39].

### 4.4 Construction of Spatial Adjacency Graph

In order to construct the spatial adjacency graph of the GATs encoder, we constructed the Adjacency matrix $A\in R^{n\times n}$, (n represents the number of spots, *A_ij_* = 1 indicates that the geometric distance between j and i is less than r)

For different detection positions of multiple omics platforms, such as the six‐domain structure of 10x Visium and the four‐neighborhood structure of MALDI‐MSI, we constructed the Adjacency matrix $A\in R^{n\times m}$, (n, m represents the number of spots on different spatial‐omics platforms), forming the spatial adjacency relationship between two spatial‐omics. For each spot, we used scikit‐learn [39] to calculate the k nearest Euclidean distances as neighbors.

### 4.5 AgaeSMO Model Architecture

#### 4.5.1 GATs Encoder

Let expression matrix $H\in R^{n\times f}$, *n* represents the number of spots of spatial omics. *f* represents the features of the spot, usually a gene, ATAC site, or protein. let adjacent matrix $A\in R^{n\times n}$, *A* represents the adjacency relationship of spots in spatial omics. $H=\{\vec{h_{1}},\vec{h_{2}},\vec{h_{3}},\vec{h_{4}}\text{…}\vec{h_{n}}\}$ each $\vec{h_{i}}$ represents expression of spot, *H* will multiply with a parameter $W_{1}\in R^{f\times f_{1}}$, and generate *H*′ which is low dimensional representation of spot. Then 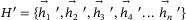, every two spots were calculated to get the attention weights as follows:

(2)

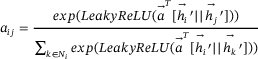




Attention weights matrix $a\in R^{n\times n}$, was masked by adjacent matrix $A\in R^{n\times n}$, and determined the weight of information gathered from adjacent spot for each spot. Then, *H*′ was multiplied by attention weight *a* to complete the encoding. Here we set *n* as the number of spots, *f* as the number of highly variable genes selected. *f*_1_ = 30 was chosen as the latent variable dimension

#### 4.5.2 GATs Decoder

Let latent variable matrix $H\in R^{n\times f}$, *n* represents the number of spots of spatial omics. *f* represents the latent variable dimension of the spot. let adjacent matrix $A\in R^{n\times n}$, *A* represents the adjacency relationship of spots in spatial omics.$\;H=\{\vec{h_{1}},\vec{h_{2}},\vec{h_{3}},\vec{h_{4}}\text{…}\vec{h_{n}}\}$ each $\vec{h_{i}}$ represents low dimensional representation of spot, *H* multiplies with a parameter $W_{1}\in R^{f\times f_{1}}$, and generates *H*′ which is reconstructed gene expression of spot. Then 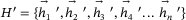, every two spots were calculated the attention weights as Formula 2. Attention weights matrix $a\in R^{n\times n}$, was masked by adjacent matrix $A\in R^{n\times n}$, and was determined the weight of information gathered from adjacent spot for each spot. Then, *H*′ was multiplied by attention weight *a* to complete the decoding. Here we set *n* as the number of spots, *f* _1_as the number of highly variable genes selected. *f* = 30 was chosen as the latent variable dimension.

#### 4.5.3 Modality Fusion Attention Layer

In order to integrate the information from two graphs, we built a modal fusion attention module. Let embedding matrix $H_{1}\in \;R^{n\times f}=\left\{\vec{h_{1}^{1}},\vec{h_{2}^{1}},\vec{h_{3}^{1}},\vec{h_{4}^{1}}\text{…}\vec{h_{n}^{1}}\right\}$ and $\;H_{2}\in \;R^{n\times f}=\left\{\vec{h_{1}^{2}},\vec{h_{2}^{2}},\vec{h_{3}^{2}},\vec{h_{4}^{2}}\text{…}\vec{h_{n}^{2}}\right\}$, Each vector $\vec{h}\in R^{f\times 1}$ in *H*_1_ and *H*_2 _ represents spot embedding, which was multiplied by the same of parameters to obtain attention weights, attention coefficient fellows:

(3) $$e_{i,t}=w^{T}\;\tanh\left(Wh_{i,t}\right)$$

*t* represents the number of graphs, *i* represents the number of spot, parameter $W\in R^{f\times f}$, $w\in R^{f\times 1}$. Each *e* of $\vec{h}$ forms an attention coefficient matrix. The coefficient matrices of *H*_1_ and *H*_2_ was concatenated and applied softmax function:

(4) $$a_{i,t}=\frac{exp\left(e_{i,t}\right)}{\sum_{t}exp\left(e_{i,t}\right)}$$

Then obtain modal attention weights $A=\left[a_{i,t}\right]\in R^{n\times 2}$. $H_{3}\in \;R^{n\times f}=\left\{\vec{h_{1}},\vec{h_{2}},\vec{h_{3}},\vec{h_{4}}\text{…}\vec{h_{n}}\right\}$, each $\vec{\;h_{i}}$ fellows:

(5) $$\vec{h_{i}}=a_{1,i}\;\vec{h_{i}^{1}}+a_{2,i}\vec{h_{i}^{2}}$$

$H_{3}\in R^{n\times f}$ represents the spot embedding after the fusion of two expressions. *H*_1_ and *H*_2_ could be Encoded Spatial Graph and feature Graph or Encoded Graph of two modality Embedding.

#### 4.5.4 Graph Cross‐Attention Layer

Let spatial transcriptome embedding matrix $H_{1}\in \;R^{n\times k}=\{\vec{h_{1}^{1}},\vec{h_{2}^{1}},\vec{h_{3}^{1}},\vec{h_{4}^{1}}\text{…}\vec{h_{n}^{1}}\}$, n represents the number of spots, and k represents the latent representation dimension of spots. Let Spatial metabolome embedding matrix $H_{2}\in \;R^{m\times k}=\{\vec{h_{1}^{2}},\vec{h_{2}^{2}},\vec{h_{3}^{2}},\vec{h_{4}^{2}}\text{…}\vec{h_{m}^{2}}\}$, m represents the number of spots, and k represents the latent representation dimension of spots. *H*_1_, *H*_2_ are the potential representations of the modality after passing through the GATs encoder. Adjacency matrix *A* ∈ {0,  1}^*n*×*m*^. For each $\vec{h_{i}^{1}}$, matrix A records k spots that are spatially adjacent to it. Every two spot will be calculated attention weights follows:

(6) $$a_{ij}=\frac{\exp(LeakyReLU({\vec{a}}^{T}[\vec{h_{i}^{1}}\left|\right|\vec{h_{j}^{2}}]))}{\sum_{k\in N_{i}}exp(LeakyReLU({\vec{a}}^{T}[\vec{h_{i}^{1}}\left|\right|\vec{h_{k}^{2}}]))}$$

Attention weights matrix *a*  ∈  *R* ^*n*×*m*^ will be masked by adjacent matrix *A*. Then, *H*_2_ will be multiply Attention weights matrix *a* to generate *H*′_2_  ∈  *R* ^*n*×*k*^, which has the same tensor shape as *H*_1_

#### 4.5.5 Loss Function

AgaeSMO uses loss function follows:

(7) $$\begin{array}{ccc} Loss & = & w_{rcon}^{1}\;L_{rcon}^{1,spatial}+w_{rcon}^{2}L_{rcon}^{2,spatial}+w_{corr}^{1}L_{\mathrm{corr}}^{1,spatial}\\ & & +\;w_{corr}^{2}L_{\mathrm{corr}}^{2,spatial} \end{array}$$

$L_{rcon}^{1,spatial}$ and $L_{rcon}^{2,spatial}$ represents reconstruct loss. For each one, let expression matrix $H\in R^{n\times f}$ and $H_{rcon}\in R^{n\times f}$ as reconstruct feature *H* and *H_rcon_* will be calculate SCE loss fellows:

(8) $$L_{sce}=\frac{1}{\left|\tilde{V}\right|}\sum_{v_{i}\in \tilde{V}}{\left(1-\frac{x_{i}^{T}z_{i}}{\left|\left|x_{i}\left|\left|\cdot \right|\right|z_{i}\right|\right|}\right)}^{\gamma }$$

$L_{\mathrm{corr}}^{1,spatial}\;\mathrm{and}\;L_{\mathrm{corr}}^{2,spatial}$ represents correspondence loss, to ensure that the reconstructed features and the latent representations of modalities are consistent. Let expression matrix $H\in R^{n\times f}$, reconstruct feature $H_{rcon}\in R^{n\times f}$ and attention weight *a*  ∈  *R* ^*n*×*m*^, low‐dimension representations fellows:$\;H^{\prime }\in \;R^{n\times f}=\sigma \left(aHW\right)$, *H*′_*rcon*_ =  σ(*aH_recon_W*). Then, *H*′_*rcon*_ and *H*′ was calculated as SCE loss in the same way as the reconstruct loss. $w_{rcon}^{1}$, $w_{rcon}^{2}$, $w_{corr}^{1}$ and $w_{corr}^{2}$ are the weight factors for the loss function. By default, they are all set to 1, and can be adjusted in the training function.

### 4.6 Model Hyper‐Parameters and Training Details

Due to the differences in technical platforms, the model hyper‐parameters used for data on each technical platform can be found in Table S3.

### 4.7 Clustering of Latent Variable

After training AgaeSMO, we extracted the low‐dimensional fusion representations and used mclust [40] for unsupervised clustering. Mclust was implemented through the mclust‐R package.

### 4.8 Benchmarking Tests of AgaeSMO With Baseline Methods

For spatial domain clustering, we benchmarked AgaeSMO against SpatialGlue [9], TotalVI [14], MultiVI [15], MOFA+ [16], SoFusion [12], SpaMV [10], 3d ‐ OT [11] for multi‐omics integration, and compared it with STAGATE [19], DeepST [24], SpaGCN [23], STMGraph [20] for spatial‐transcriptome. We followed the official tutorials for implementation for each baseline method. The default parameters can be found:

Spatial Glue: https://github.com/JinmiaoChenLab/SpatialGlue
COSMOS: https://github.com/Lin‐Xu‐lab/COSMOS
TotalVI: https://docs.scvi‐tools.org/en/stable/user_guide/models/totalvi.html
MultiVI: https://docs.scvi‐tools.org/en/stable/user_guide/models/multivi.html
MOFA+: https://biofam.github.io/MOFA2/installation.html
STAGATE: https://github.com/QIFEIDKN/STAGATE_pyG
DeepST: https://github.com/JiangBioLab/DeepST
SpaGCN: https://github.com/jianhuupenn/SpaGCN
STMGraph: https://github.com/binbin‐coder/STMGraph
3d‐OT: https://3d‐ot.readthedocs.io/en/latest
SoFusion: https://github.com/sunxue‐yy/soFusion
SpaMV: https://github.com/ericcombiolab/SpaMV

### 4.9 Evaluation Metrics

We compared AgaeSMO with existing methods and evaluated by clustering metrics:

ARI (adjusted rand index):

$$\begin{array}{c} \mathrm{ARI}=\frac{\sum_{i,j}\left(\begin{array}{c} n_{ij}\\ 2 \end{array}\right)-\frac{\left[\sum_{i}\left(\begin{array}{c} a_{i}\\ 2 \end{array}\right)\sum_{j}\left(\begin{array}{c} b_{j}\\ 2 \end{array}\right)\right]}{\left(\begin{array}{c} n\\ 2 \end{array}\right)}}{\frac{1}{2}\left[\sum_{i}\left(\begin{array}{c} a_{i}\\ 2 \end{array}\right)+\sum_{j}\left(\begin{array}{c} a_{j}\\ 2 \end{array}\right)\right]-\frac{\left[\sum_{i}\left(\begin{array}{c} a_{i}\\ 2 \end{array}\right)\sum_{j}\left(\begin{array}{c} b_{j}\\ 2 \end{array}\right)\right]}{\left(\begin{array}{c} n\\ 2 \end{array}\right)}} \end{array}$$

AMI (adjusted mutual information):

$$\begin{array}{c} \mathrm{AMI}=\frac{\mathrm{MI}\left(U,V\right)-E\left[\mathrm{MI}\left(U,V\right)\right]}{\max\left(\mathrm{MI}\left(U,U\right),\;\mathrm{MI}\left(V,V\right)\right)-E\left[\mathrm{MI}\left(U,V\right)\right]} \end{array}$$

NMI (normalized mutual information):

$$\mathrm{NMI}=\frac{2\cdot \mathrm{MI}\left(U,V\right)}{H\left(U\right)+H\left(V\right)}$$

Homogeneity:

$$Homoeneity=1-\frac{H\left(C|K\right)}{H\left(C\right)}$$

Mutual information:

$$I\left(X;Y\right)=\sum_{y\in Y}\sum_{x\in X}p\left(x,y\right)\log\left(\frac{p\left(x,y\right)}{p\left(x\right)p\left(y\right)}\right),$$

V measure:

$$V=\frac{2\cdot h\cdot c}{h+c}$$

All metrics were calculated by scikit‐learn package [39].

### 4.10 Ablation Study

To systematically evaluate the contributions of core components of AgaeSMO, we performed ablation experiments. We compared AgaeSMO full model against three variants: 1) GATs modules replaced by GCN encoder‐decoder (GCN En/Decoder), 2) cross‐modal alignment module without attention (e.g. Alignment w/o Attn), and 3) feature aggregation substituted by direct simple aggregation (e.g. Concatenation). All models were trained under identical conditions, and clustering performance evaluated by ARI. This design disentangled individual effects of feature extraction, cross‐modal alignment, and feature integration in the AgaeSMO framework. Experiments were performed on human lymph node (HLN) A1 datasets and mouse brain SMA 109 B1 datasets.

### 4.11 Swapping Study

To systematically evaluate the robustness and modality dependence of integration framework, we conducted a modality swapping experiment. The input order of two modalities were swapped: “modality 1 to 2” and “modality 2 to 1,” to assess whether clustering performance biases toward the primary query modality. All other hyperparameters and network architectures were kept identical. Clustering performance was quantified by the ARI of embeddings. When spatial domain structure generated by swapped embeddings mismatching anatomical ground truth, ARI were not computable due to invalid one‐to‐one label mapping. We instead qualitatively inspected spatial domain maps to evaluate structural retention and regional distinguishability. Experiments were carried out on both SMA 109 A1 and 038 B1 datasets.

### 4.12 UMAP Analysis

We plotted the umap using *scanpy.pl.umap* function.

### 4.13 Pseudo‐Spatiotemporal Maps (pSM)

We employed the Pseudo‐spatiotemporal analysis using the adapted Gaussian kernel suggested by Haghverdi et al. [41], which implemented in the *scanpy.tl.diffmap* function.

### 4.14 Identifying Spatial Differentially Expressed Genes (SDEGs)

We used the Wilcoxon test implemented in SCANPY [38] package to identify spatial differentially expressed genes (SDEGs) for each spatial domain.

### 4.15 Calculating Correlation Between pSM and Gene Expression

We used the Pearson correlation to measure the correlation between gene expression and pSM, and selected the results with a *p*‐value < 0.05. This was implemented using scikit‐learn [39].

## Author Contributions

X.M. conceived and designed the project. K.L. and X.M. developed the AgaeMSO algorithm and performed the data analysis. K.L., J.S., H.Z., Y.S., M.Q., Y.L., T.W., Z.C., Y.W. performed the bioinformatics analyses. K.L. generated GitHub page for AgaeMSO software. X.M. acquired the funding. K.L. and X.M. wrote and revised the manuscript. All authors have read, revised, and approved the final manuscript.

## Conflicts of Interest

The authors declare no conflicts of interest.

## Supporting information




**Supporting File**: advs78141‐sup‐0001‐SuppMat.pdf.

## Data Availability

The data that supports the findings of this study are available in the supplementary material of this article. All datasets used in this study were obtained from previously published sources. The sources of datasets used in this study has been listed in Supplementary Table S1. The compiled datasets is also available in the Zenodo repository at https://zenodo.org/records/22934477.
