# Supplementary material for: Flexible Integration of Structurally Diverse Spatial Multi‐Omics With AgaeSMO
Source: Adv Sci (Weinh). 2026 Oct 12:e78141. Online ahead of print. doi: 10.1002/advs.78141 (PMC13658882; doi:10.1002/advs.78141)
Supplement: Supplementary file 1 [file ADVS-9999-e78141-s001.pdf]

Flexible Integration of Structurally Diverse Spatial Multi-omics with AgaeSMO

Supplementary results

Impacts of weighting factors in loss function on attention weights of modalities

We investigated the effects of weighting factors (W/F) in loss function on attention weights of modalities across different datasets. Because our model set the weighing factor (W/F ratio) of loss function as the weights of reconstruction loss and correspondence loss for modality 1 versus 2. When the reconstruction loss/correspondence loss of one modality is substantially larger than that of the other, the attention weights tend to allow more information from former modality to pass through, since the embeddings are aggregated according to the attention weights.

1) Simulation datasets

We conducted experiments on simulated data with seven treatments for W/F ratio of RNA and Protein: (1:1:1:1), (16:1:16:1), (4:1:4:1), (1.5:1:1.5:1), (1:4:1:4), (1:16:1:16), and (1:64:1:64) (**Supplementary Figure S20**). It showed that when W/F ratio are gradually decreased (while those of the Protein reconstruction loss are increased), the attention weights progressively become dominant. This occurs because, when the weight of a modality reconstruction loss increases, the gradient descent process treats the reconstruction of that modality as more important, thereby allocating more attention weight to it. At the W/F = (1:4:1:4), the attention weights remain dominated by RNA at all clusters. It is not until the W/F = (1:16:1:16) that the Protein modality becomes dominant (**Supplementary Figure S20**).

2) Experimental datasets

We also conducted experiments on the high-resolution mouse thymus 1 and 2 datasets, with W/F ratios set to (1:1:1:1), (4:1:4:1), (2:1:2:1), (1:2:1:2), and (1:4:1:4), respectively (**Supplementary Figure S21**). In the mouse thymus 1 datasets, when W/F = (4:1:4:1) and (2:1:2:1), the model is dominated by the RNA modality, accompanied by increased clustering noise. In contrast, the clustering performance at W/F = (1:2:1:2) and (1:1:1:1) was similar, while at W/F = (1:4:1:4), the model was dominated by the protein modality. In the mouse thymus 2 datasets, only marginal differences were observed among W/F = (2:1:2:1), (1:1:1:1), and (1:2:1:2), despite the fact that these conditions were dominated by different modalities.

Our above experiments demonstrated that the weighting factors in the loss function influence the variation of modality attention weights and consequently affect clustering outcomes. Although the exact pattern of these variations remains unclear, satisfactory clustering performance can still be achieved if the modality attention weights do not differ substantially across modalities. This permissible degree of difference may vary depending on specific datasets.

Impacts of weighting factors in loss function on spatial domain clustering

We investigated the effects of weighting factors (W/F) in loss function on spatial domain clustering across different datasets.

1) Simulation datasets

We investigated the effects of the four weighting factors in the loss function on modality integration across five simulated datasets (**Supplementary Figure S22a**). We designed seven treatments for W/F ratios: (1:1:1:1), (16:1:16:1), (4:1:4:1), (1.5:1:1.5:1), (1:4:1:4), (1:16:1:16), and (1:64:1:64). These ratios correspond to progressively increasing reconstruction and correspondence losses for the protein modality. When the W/F ratio was 1:1:1:1, all ARI values exceeded 0.99. Increasing the weights of the RNA reconstruction loss and correspondence loss to 16:1:16:1 and 4:1:4:1 yielded ARI values ranging between 0.96 and 0.99 (for 16:1:16:1) and between 0.98 and 0.99 (for 4:1:4:1). When the W/F= (1.5:1:1.5:1), only Sim 4 exhibited an ARI below 0.99. Furthermore, when the ratio scale differences became lager, the ARI value tends to stabilize (remaining greater than 0.99). Even when the modality weights are dominated by a single modality (within a certain range), the clustering performance remains largely unaffected. Our experiments demonstrate that W/F indeed improves model clustering performance within a certain range. Importantly, the 1:1:1:1 ratio adopted in our main experiments not only falls within this range but also outperforms all existing state-of-the-art approaches.

2) Experimental datasets

We further investigated the effects of the four weighting factors on SMA 109 A1 Datasets (**Supplementary Figure S22b**). We designed five treatments for W/F ratios: (1:1:1:1), (4:1:4:1), (2:1:2:1), (1:2:1:2) and (1:4:1:4). After adjusting the W/F ratio, AgaeSMO still successfully distinguished between CP-intact and CP-lesioned conditions. Only at a W/F ratio of 4:1:4:1, the ARI fall slightly below that of STAGATE (with a difference of approximately 0.003), while at all other W/F ratios, AgaeSMO outperformed the other methods. We also investigated the effects of the four weighting factors on mouse thymus 2 datasets (**Supplementary Figure S22c**). We designed five treatments for W/F ratios: (1:1:1:1), (4:1:4:1), (2:1:2:1), (1:2:1:2) and (1:4:1:4). All the primary clustering results also showed minor differences in noise levels under different W/F ratios.

Our above experiments showed that, within a certain range, the weighting factor of the loss function has little effect on the clustering performance. This range varies across datasets. For example, a 64-fold difference is required to produce a noticeable effect on simulated data, whereas a 4-fold difference is sufficient on real data, and the range typically does not exceed 10-fold. Our AgaeSMO software defines the weighting factors for the loss terms as adjustable parameters, with a default setting of (1:1:1:1). Users may tune these parameters according to the characteristics of specific dataset to achieve optimal clustering performance. However, even with default (1:1:1:1) weighting factor, the software already yields satisfactory clustering results in the datasets we tested in main text, that outperforms existing state-of-the-art approaches.

Supplementary Tables

Supplementary Table S1. Datasets used in this paper.

Datasets	Tissue	Data Source	Dataset Dimensions	Protocol
Simulated datasets 1-5	Simulated	https://zenodo.org/records/10362607	Modality1:1296*1000 Modality2:1296*100	
Human Lymph Node A1	Human Lymph Node	https://drive.google.com/drive/folders/1RlU3JmHg_LZM1d-o6QORvykYPoulWWMI	RNA:3484*18085 Protein:3484*31	10x Visium (RNA-protein)
P22 mouse brain coronal sections	Mouse brain	https://zenodo.org/records/10362607	RNA:9215*22914 ATAC：9215*121068	ATAC–RNA-seq and CUT&Tag-RNA-seq
SMA V11L12-109_A1	Mouse brain	https://data.mendeley.com/datasets/w7nw4km7xd/1	RNA:2675*32285 MSI:2438* 1538	10x Visium MALDI MSI
SMA V11L12-109_B1	Mouse brain	https://data.mendeley.com/datasets/w7nw4km7xd/1	RNA:3120*32285 MSI:3005*1538	10x Visium MALDI MSI
SMA V11L12-109_C1	Mouse brain	https://data.mendeley.com/datasets/w7nw4km7xd/1	RNA:2918*32285 MSI:3040*1538	10x Visium MALDI MSI
Mouse Thymus 1	Mouse Thymus	https://drive.google.com/drive/folders/1RlU3JmHg_LZM1d-o6QORvykYPoulWWMI	RNA:4697*23622 RNA downsampled: 1216*23622 ADT:4697*51 ADT downsampled: 1213*51	Stereo-CITE-Seq
Mouse Thymus 2	Mouse Thymus	https://drive.google.com/drive/folders/1RlU3JmHg_LZM1d-o6QORvykYPoulWWMI	RNA:4253*23529 RNA downsampled: 1110*23529 ADT:4253*19 ADT downsampled: 1097*19	Stereo-CITE-Seq
Human breast Cancer datasets	Human breast Cancer	https://cf.10xgenomics.com/samples/spatial-exp/1.1.0/V1_Breast_Cancer_Block_A_Section_1/V1_Breast_Cancer_Block_A_Section_1_web_summary.html	RNA:3798 * 36601	10x Visium
orchid flower	Slide 1	https://academic.oup.com/nar/article/50/17/9724/6696353#supplementary-data	RNA:2379 × 28903	10x Visium

Supplementary Table S2. Details of Data Preprocessing.

Datasets	Preprocessing	
Simulated datasets 1-5	RNA modality:<br>sc.pp.filter_genes(min_cells=10)<br>sc.pp.highly_variable_genes(n_top_genes=100)<br>sc.pp.normalize_total(target_sum=1e4)<br>sc.pp.log1p()<br>sc.pp.scale()	Protein modality:<br>clr_normalize_each_cell()<br>sc.pp.scale()
Human Lymph Node A1	RNA modality:<br>sc.pp.filter_genes(min_cells=10)<br>sc.pp.highly_variable_genes(n_top_genes=3000)<br>sc.pp.normalize_total(target_sum=1e4)<br>sc.pp.log1p()<br>sc.pp.scale()	Protein modality:<br>clr_normalize_each_cell()<br>sc.pp.scale()<br>Then, the data of Protein modality will undergo PCA dimensionality reduction to 30 dimensions.
P22 mouse brain coronal sections	RNA modality:<br>sc.pp.filter_genes(min_cells=10)<br>sc.pp.highly_variable_genes(n_top_genes=3000)<br>sc.pp.normalize_total(target_sum=1e4)<br>sc.pp.log1p()<br>sc.pp.scale()<br>3000 highly_variable_genes will be used as the input for the model.	ATAC modality:<br>sc.pp.highly_variable_genes(n_top_genes=3000)<br>clr_normalize_each_cell()<br>sc.pp.normalize_total(target_sum=1e4)<br>sc.pp.log1p()<br>sc.pp.scale()<br>3000 highly_variable_genes will be used as the input for the model.
SMA V11L12-109_A1<br>SMA V11L12-109_B1<br>SMA V11L12-109_C1	RNA modality:<br>sc.pp.filter_genes(min_cells=10)<br>sc.pp.highly_variable_genes(n_top_genes=2000)<br>sc.pp.normalize_total(target_sum=1e4)<br>sc.pp.log1p()	Metabolome modality:<br>sc.pp.highly_variable_genes(n_top_genes=1000)<br>sc.pp.log1p()
Mouse Thymus 1-2	RNA modality:<br>sc.pp.filter_genes(min_cells=10)<br>sc.pp.highly_variable_genes(n_top_genes=3000)<br>sc.pp.normalize_total(target_sum=1e4)<br>sc.pp.log1p()<br>sc.pp.scale()	Protein modality:<br>clr_normalize_each_cell()<br>sc.pp.scale()<br>Then, the data of Protein modality will undergo PCA dimensionality reduction to 30 dimensions.
Human breast Cancer datasets<br><br>Orchid flower	RNA modality:<br>sc.pp.filter_genes(min_cells=3)<br>sc.pp.highly_variable_genes(n_top_genes=3000)<br>sc.pp.normalize_total(target_sum=1e4)<br>sc.pp.log1p()<br>sc.pp.scale()	H&E modality:<br>Normalization

Supplementary Table S3. Details of Modal hyper-parameters.

Datasets	Learning rate	Weight decay	epoch	KNN	N neighbors
Simulated datasets 1-5	0.001	0	2000	4	4
Human Lymph Node A1	0.001	0	600	6	6
P22 mouse brain coronal sections	0.001	0.001	2000	4	4
SMA V11L12-109_A1	0.001	0.005	600	6	ST:6 MSI:4
SMA V11L12-109_B1	0.001	0.005	600	6	ST:6 MSI:4
SMA V11L12-109_C1	0.001	0.005	600	6	ST:6 MSI:4
Mouse Thymus 1-2 ADT+RNA	0.001	0.005	600	4	RNA rad:100 ADT rad:200
Mouse Thymus 1-2 ADT+RNA downsampled	0.001	0.005	600	4	RNA rad:250 ADT rad:100
Mouse Thymus 1-2 ADT downsampled+RNA	0.001	0.005	600	4	RNA rad:100 ADT rad:250
Human breast Cancer datasets	0.001	0	1000	1	6
Orchid flower	0.001	0	1000	1	6

Supplementary Table S4. The DEGs between Group 20/13 versus rest regions in Human Breast Cancer Datasets

Group 20 vs rest			Group 13 vs rest		
Genes	scores	*P*-values	Genes	scores	*P*-values
IGFBP5	18.719383	3.441130190182678e-78	*CRISP3*	16.326487	6.396262132966749e-60
CRISP3	18.28313	1.1276338880231952e-74	*SLITRK6*	15.34281	3.9569319969569356e-53
VTCN1	17.977137	2.943031713069984e-72	*NUPR1*	15.07807	2.2577349545796384e-51
H3F3A	17.914457	9.095334086989737e-72	*CEACAM6*	15.0170145	5.680915098814578e-51
SLITRK6	17.628918	1.4777082062988433e-69	*S100A11*	14.736623	3.750753121882903e-49
EFHD1	17.542902	6.739720099206276e-69	*PSMA6*	14.212557	7.657717297486709e-46
C6orf141	17.105043	1.360998361514009e-65	*S100A16*	14.151125	1.8379428788733952e-45
AZGP1	17.088541	1.8063972395898058e-65	*S100A13*	14.058388	6.842702174958291e-45
SERHL2	17.0622	2.8366613700983362e-65	*H3F3A*	13.890539	7.228895101514761e-44
S100A13	16.980711	1.140940675181005e-64	*IGFBP5*	13.8551235	1.1845343745239901e-43
CTTN	16.541826	1.833803849706427e-61	*SERHL2*	13.844801	1.3675988684184594e-43

Supplementary Table S5. The DEGs between Group 20 versus Group 13 in Human Breast Cancer Datasets

Names	Scores	*P*-values
APOE	11.440689	2.617965741354089e-30
RPLP1	11.180194	5.097853201874573e-29
APOC1	10.414548	2.1280678082319064e-25

Supplementary Figures

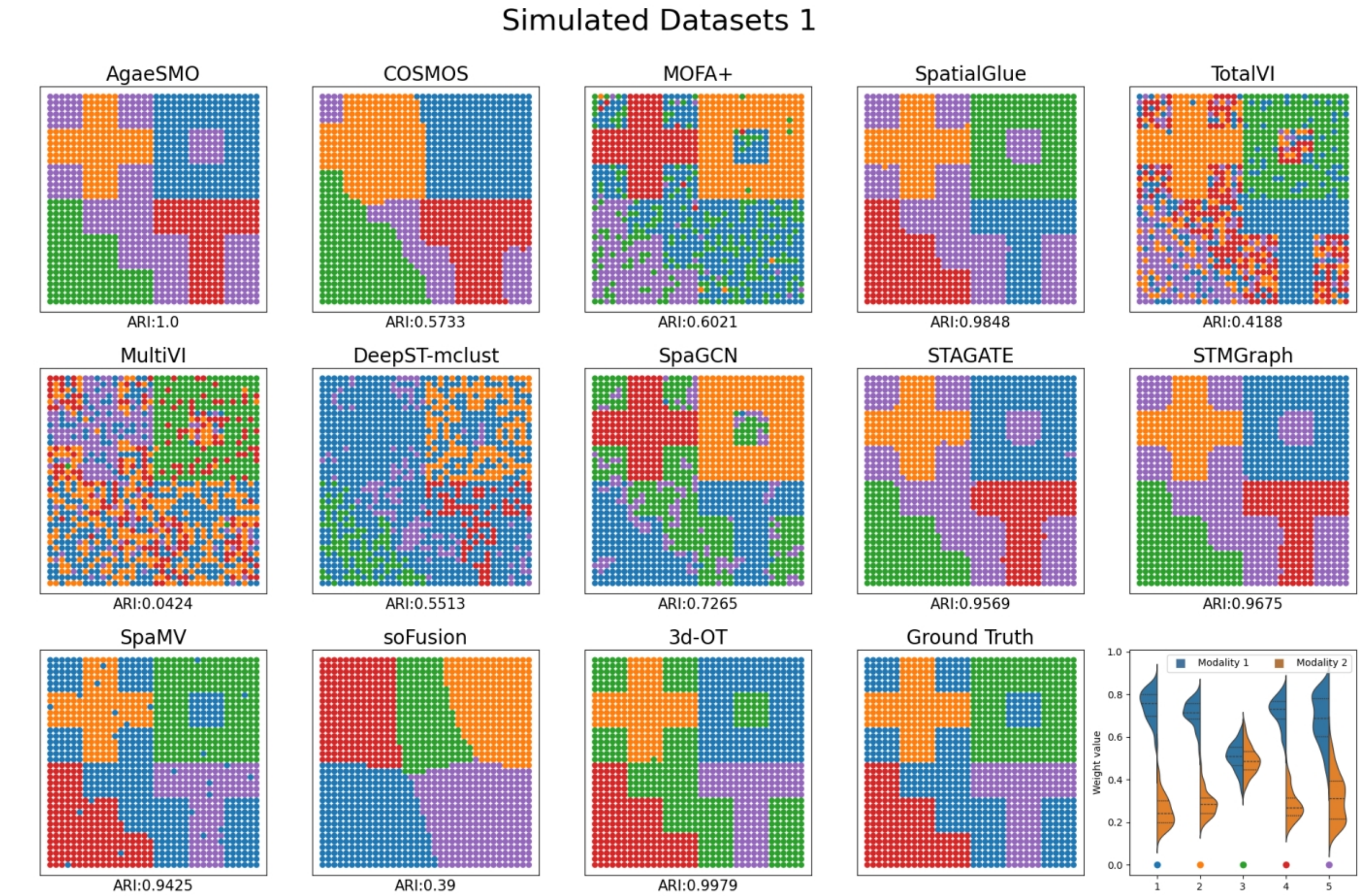

Supplementary Figure S1. Clustering performance of AgaeSMO compared with existing methods on simulated datasets 1, along with ground truth and modality weights.

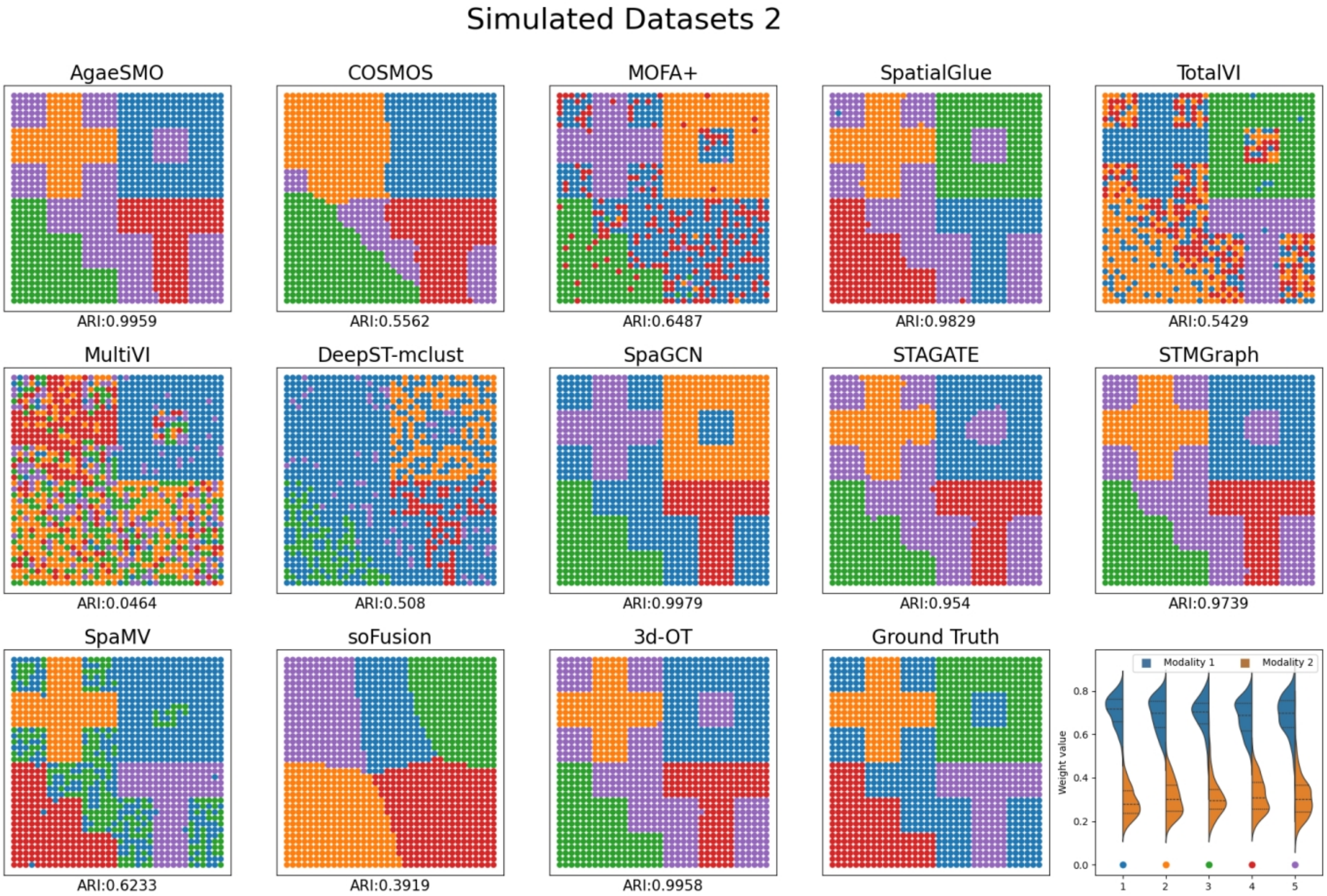

Supplementary Figure S2. Clustering performance of AgaeSMO compared with existing methods on simulated datasets 2, along with ground truth and modality weights.

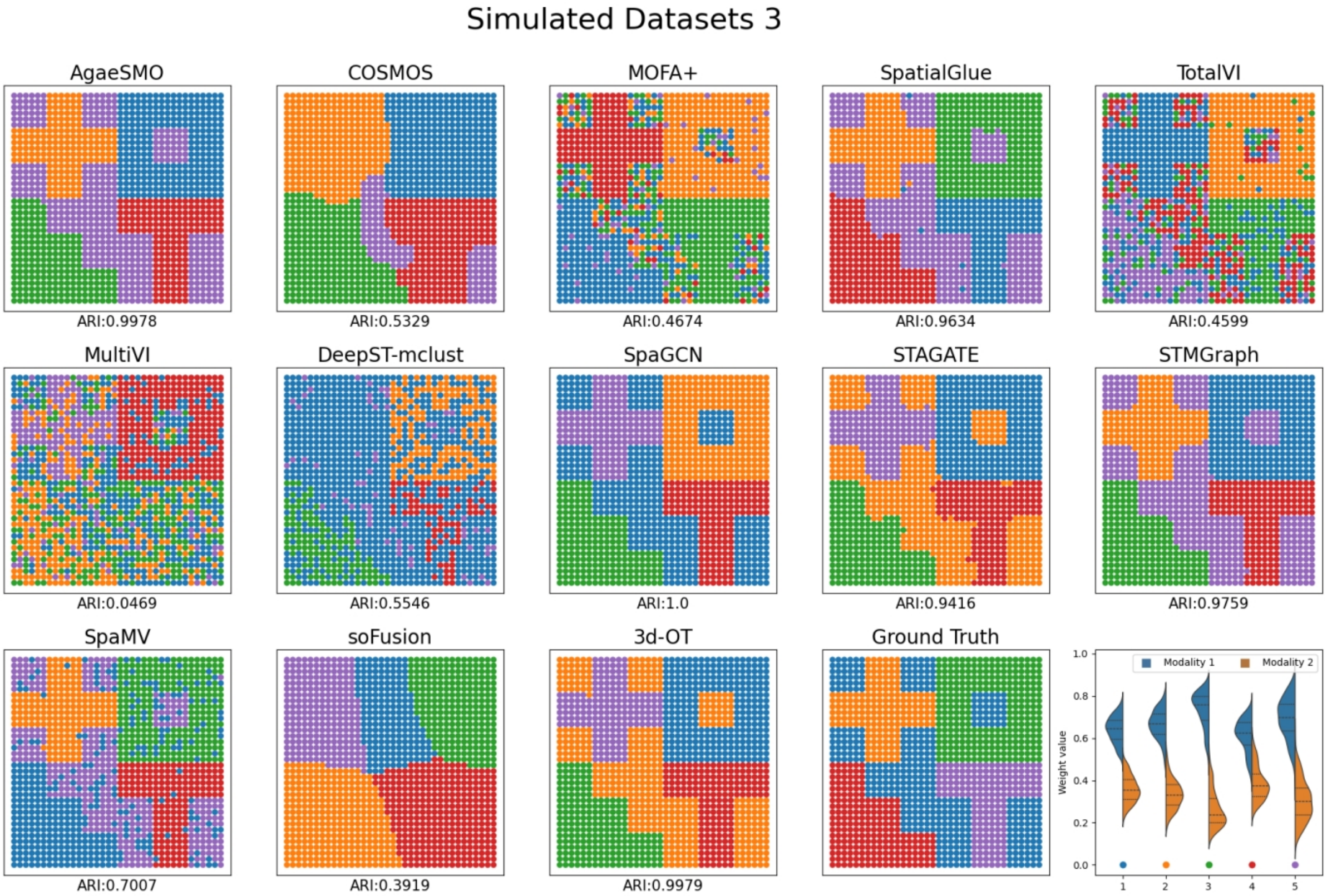

Supplementary Figure S3. Clustering performance of AgaeSMO compared with existing methods on simulated datasets 3, along with ground truth and modality weights.

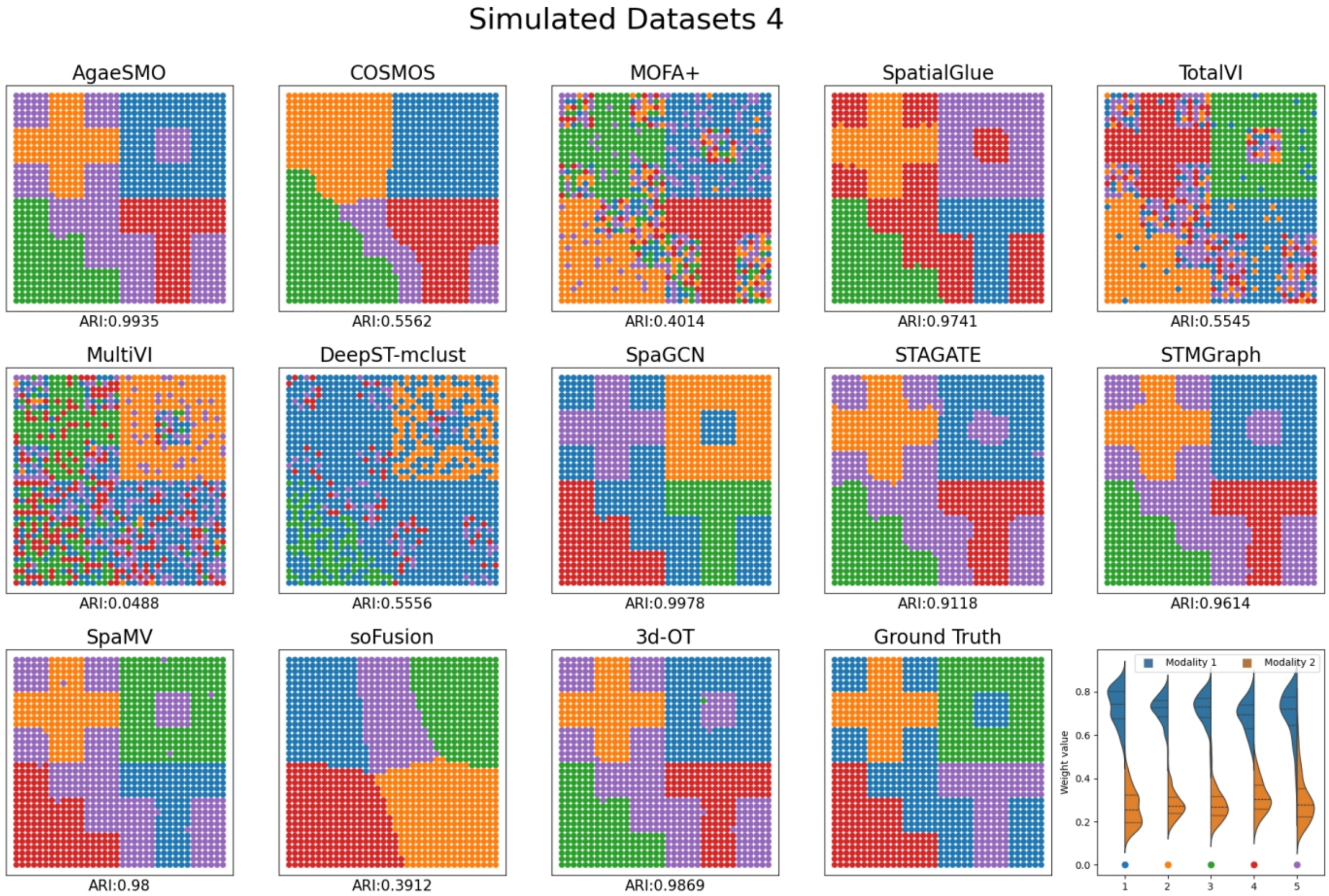

Supplementary Figure S4. Clustering performance of AgaeSMO compared with existing methods on simulated datasets 4, along with ground truth and modality weights.

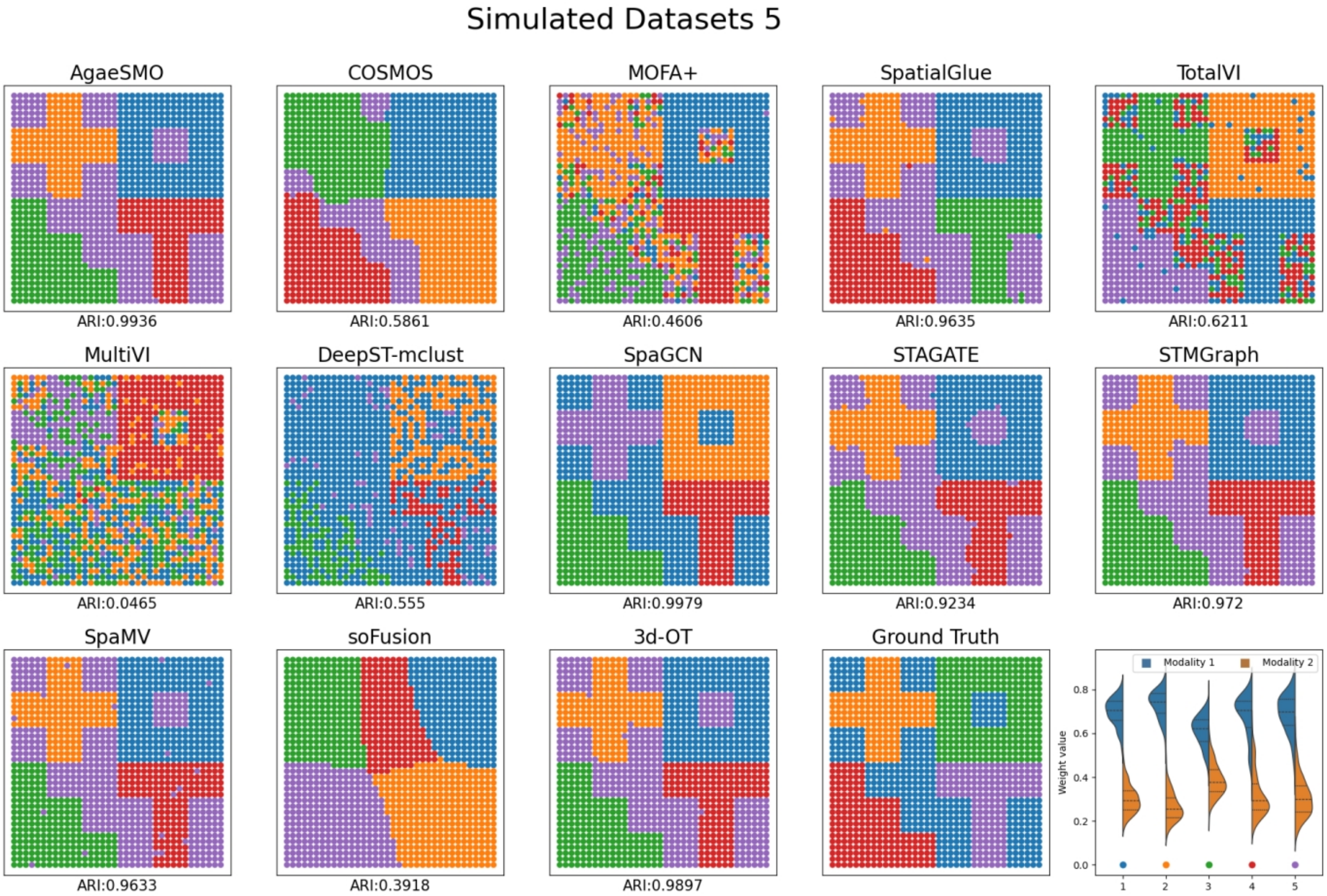

Supplementary Figure S5 Clustering performance of AgaeSMO compared with existing methods on simulated datasets 5, along with ground truth and modality weights.

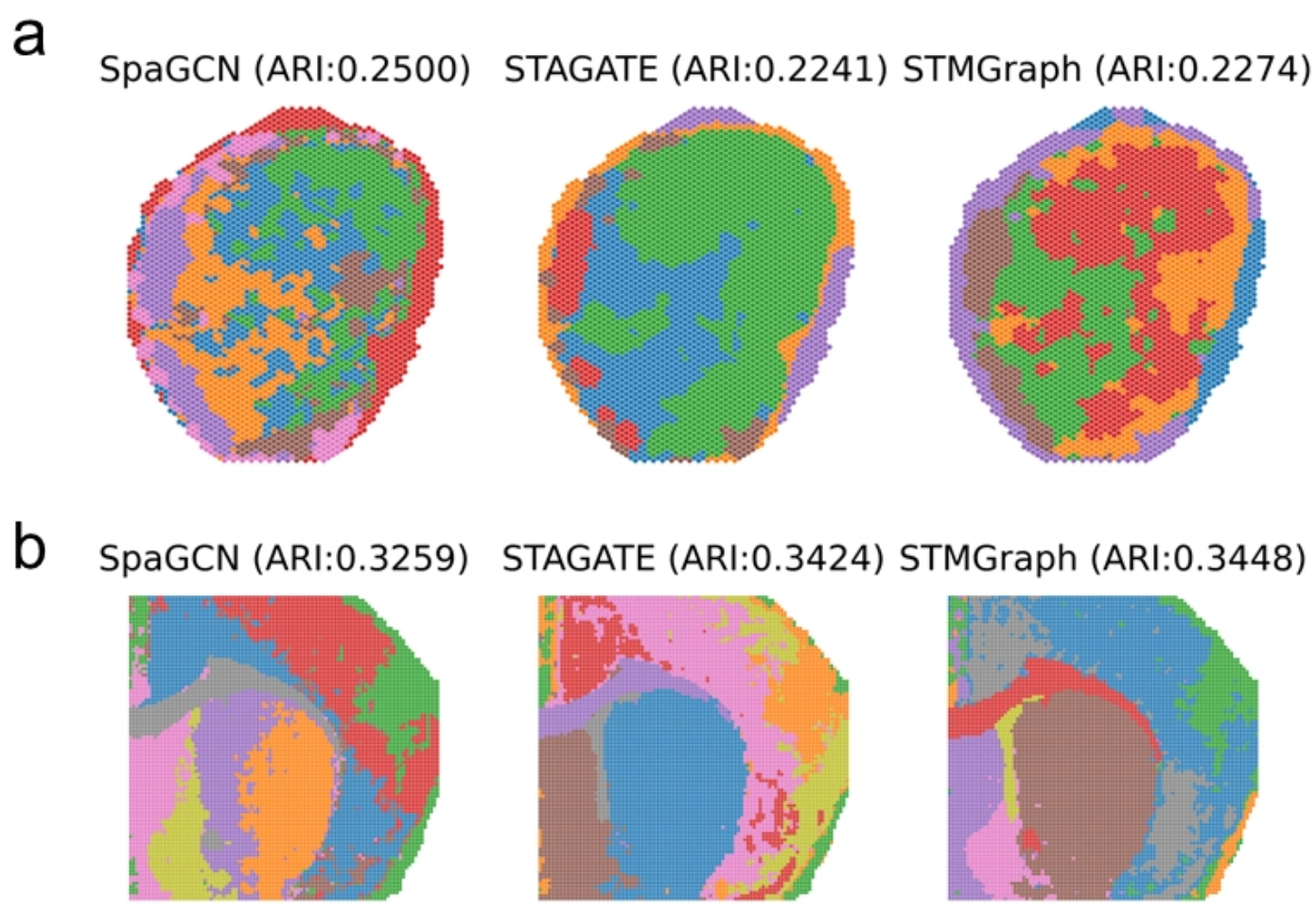

Supplementary Figure S6. Clustering results of SpaGCN, STAGATE, STMGraph on HLN A1 dataset (a) and P22 Mouse Brain dataset (b).

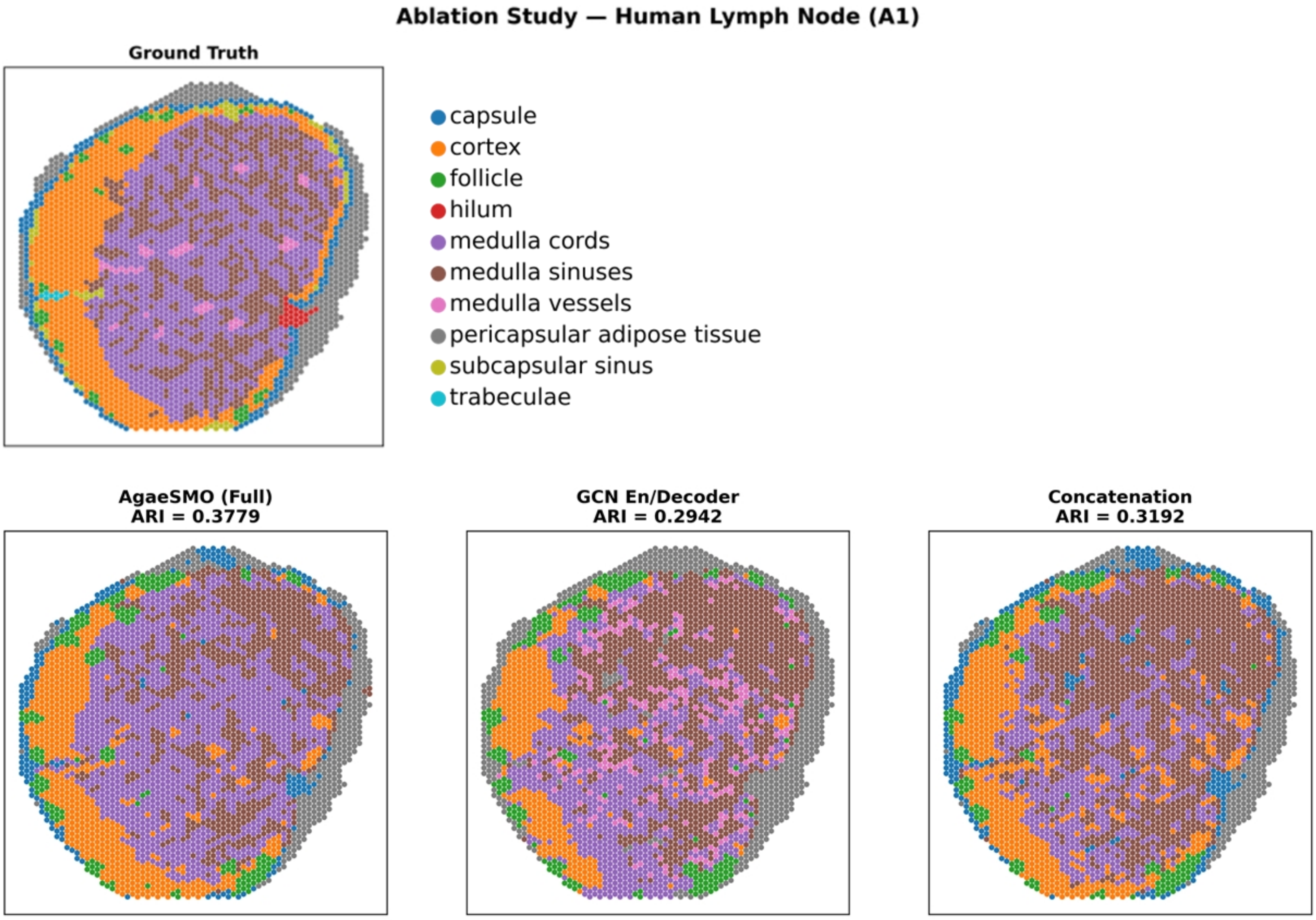

Supplementary Figure S7. Ablation study on HLN A1 dataset. AgaeSMO with GATs (e.g. AgaeSMO full) achieves the greatest improvement over with GCNs (e.g. GCN En/Decoder) in terms of model performance, while GCNs introduces more noise than GATs. The direct simple aggregation (e.g. Concatenation) could identified the capsule region, but still misidentified domain within medulla cords.

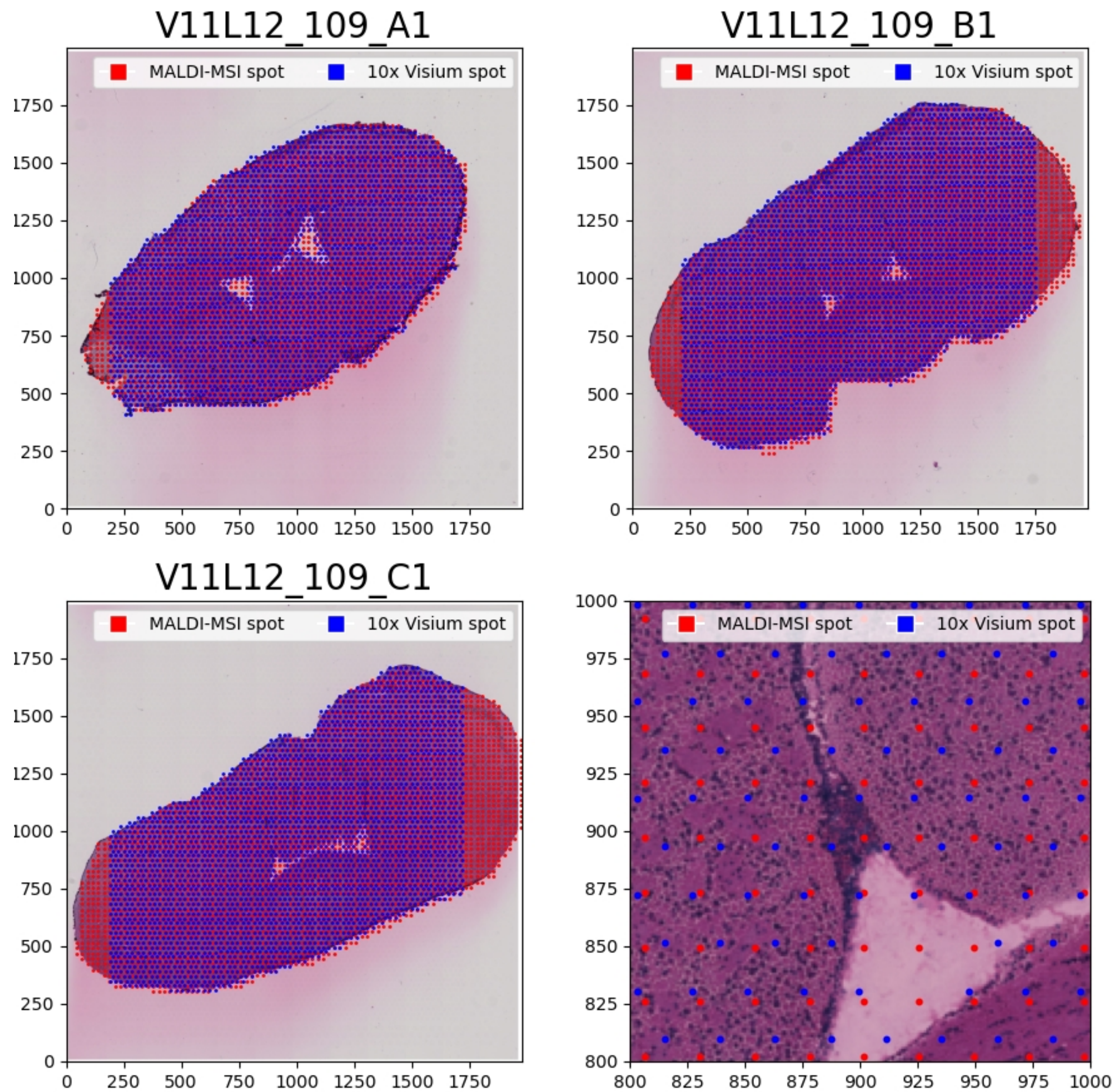

Supplementary Figure S8. The two modalities of the SMA dataset have unpaired spot positions, with spatial transcriptome is six neighborhoods structure while MALDI-MSI is four neighborhoods structure.

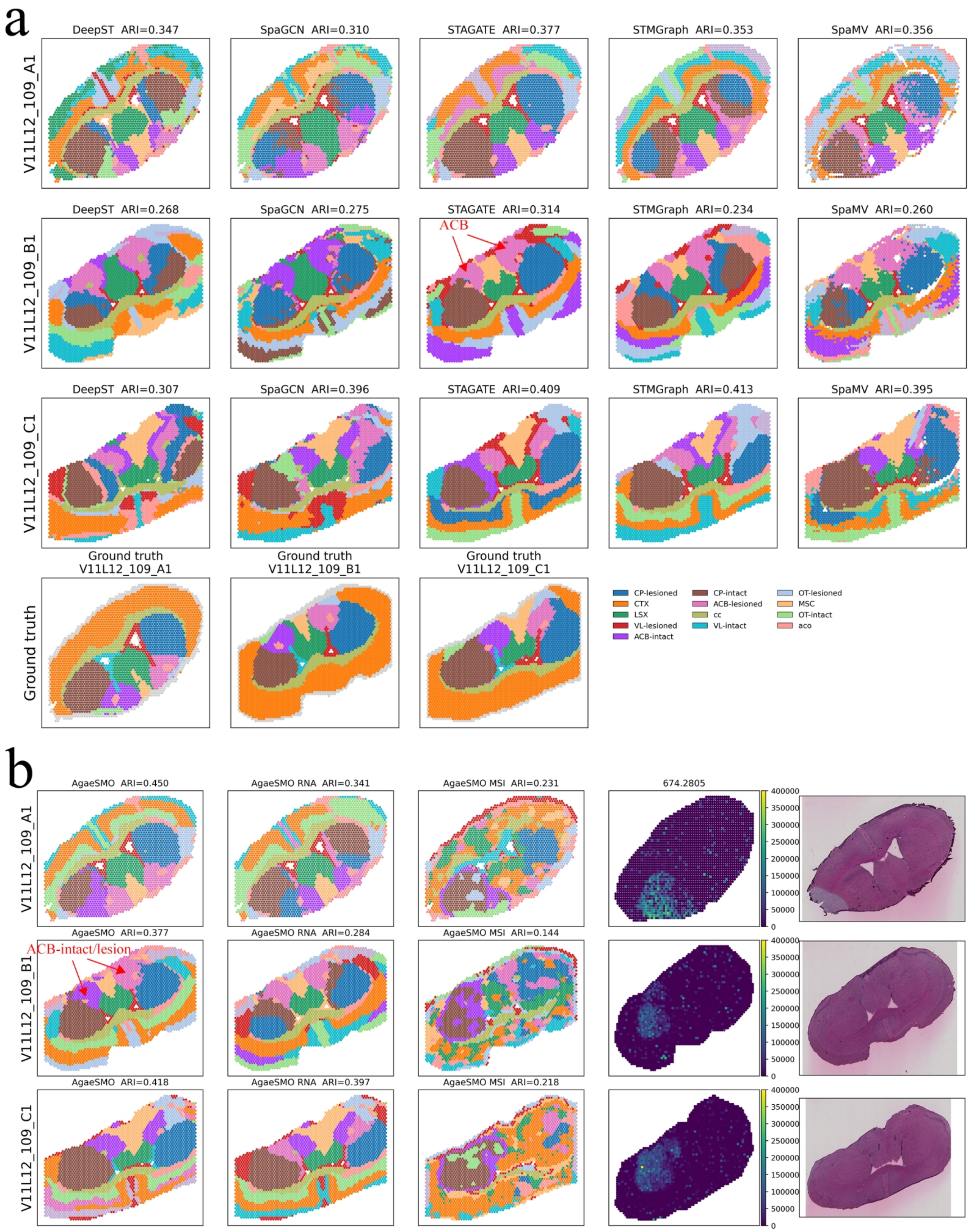

Supplementary Figure S9. Performance of AgaeSMO compared with existing methods on integration of spatial-transcriptome with other modalities. a Performance of AgaeSMO compared with existing methods on spatial-transcriptome modalities. **b** AgaeSMO integrates MALDI-MSI and spatial-transcriptome to distinguish two CP (Caudoputamen) regions with different dopamine levels.

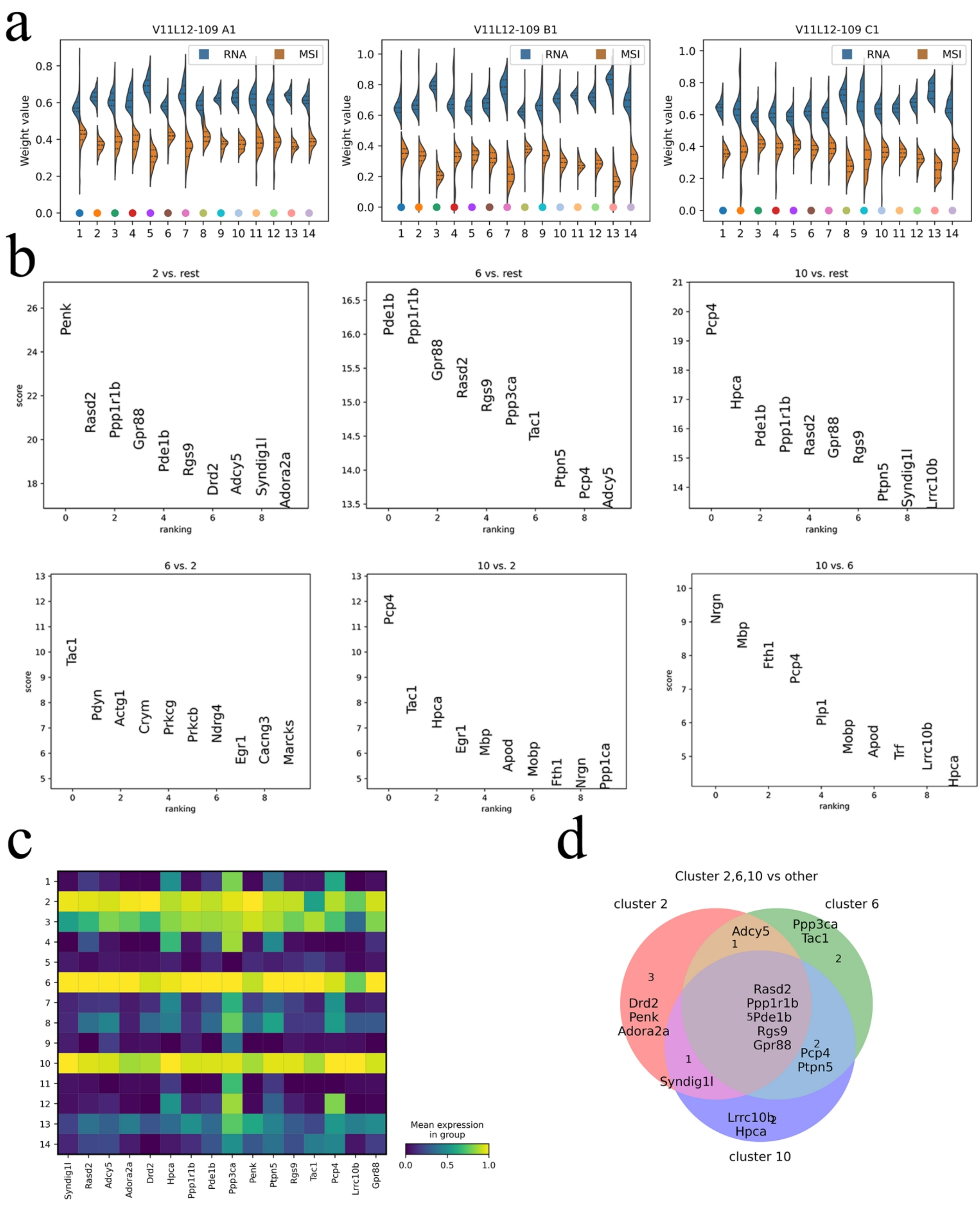

Supplementary Figure S10. Downstream analysis of 109 A1 dataset based on the clustering results of AgaeSMO. a Modality weights between RNA and MSI in SMA datasets during AgaeSMO clustering. **b** SDEGs of 109 A1 CP (Caudoputamen) cluster versus other clusters, and SDEGs of internal three CP (Caudoputamen) region (cluster 6 vs. 2, 10 vs. 2, 10 vs. 6). **c** Heatmap of SDEGs between cluster 2, 6, 10 versus other clusters. d Venn plots of 109 A1 SDEGs between cluster 2, 6, 10 versus other clusters.

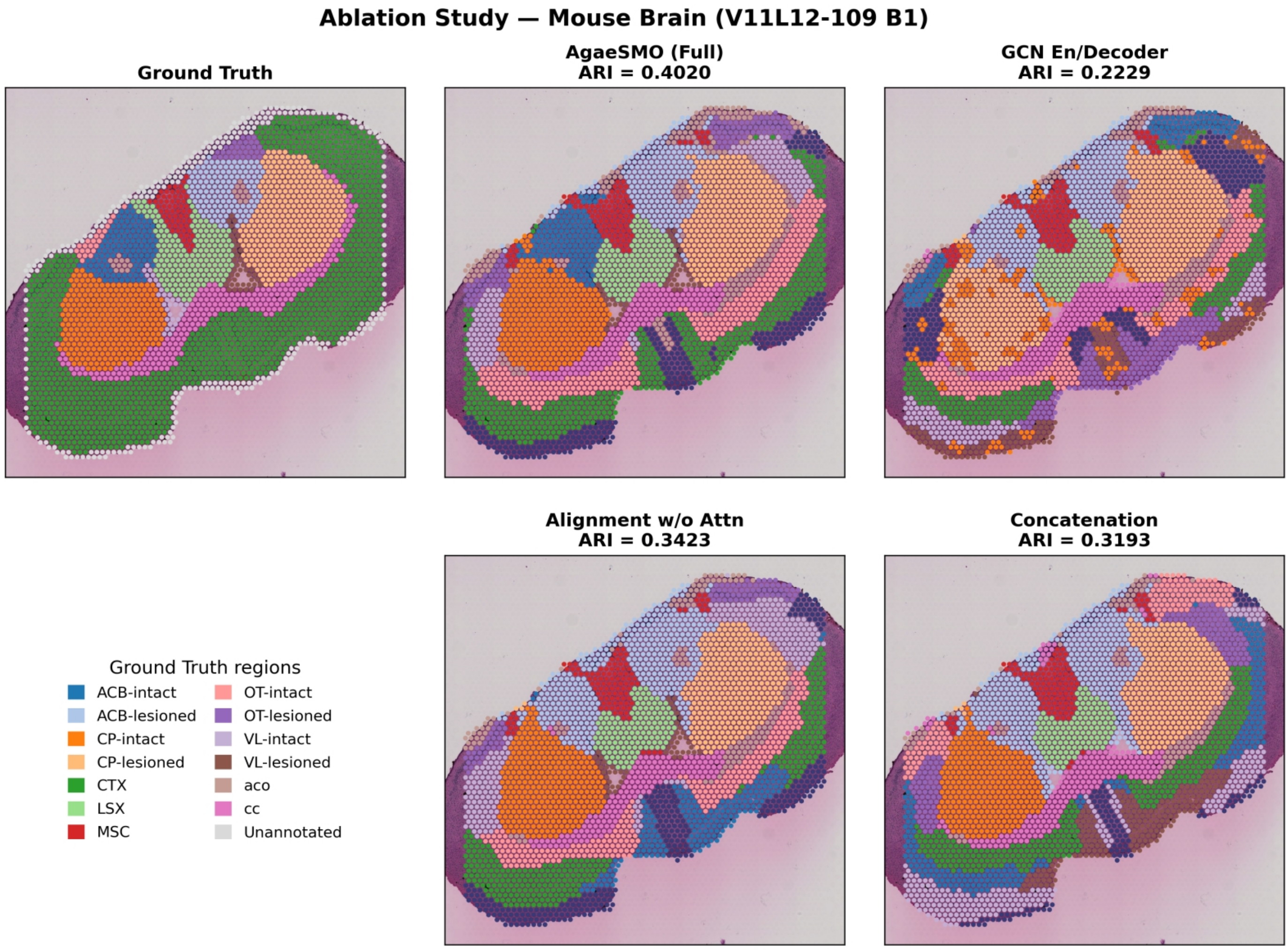

Supplementary Figure S11. Ablation study on SMA 109 B1 datasets. AgaeSMO with GATs (e.g. AgaeSMO full) achieves the greatest improvement over with GCNs (e.g. GCN En/Decoder) in terms of model performance. Cross-modal alignment module without attention (e.g. Alignment w/o Attn) and direct simple aggregation (e.g. Concatenation) could not distinguish between ACB-intact and ACB-lesioned regions. Unannotated spots are not included in the calculation of ARI.

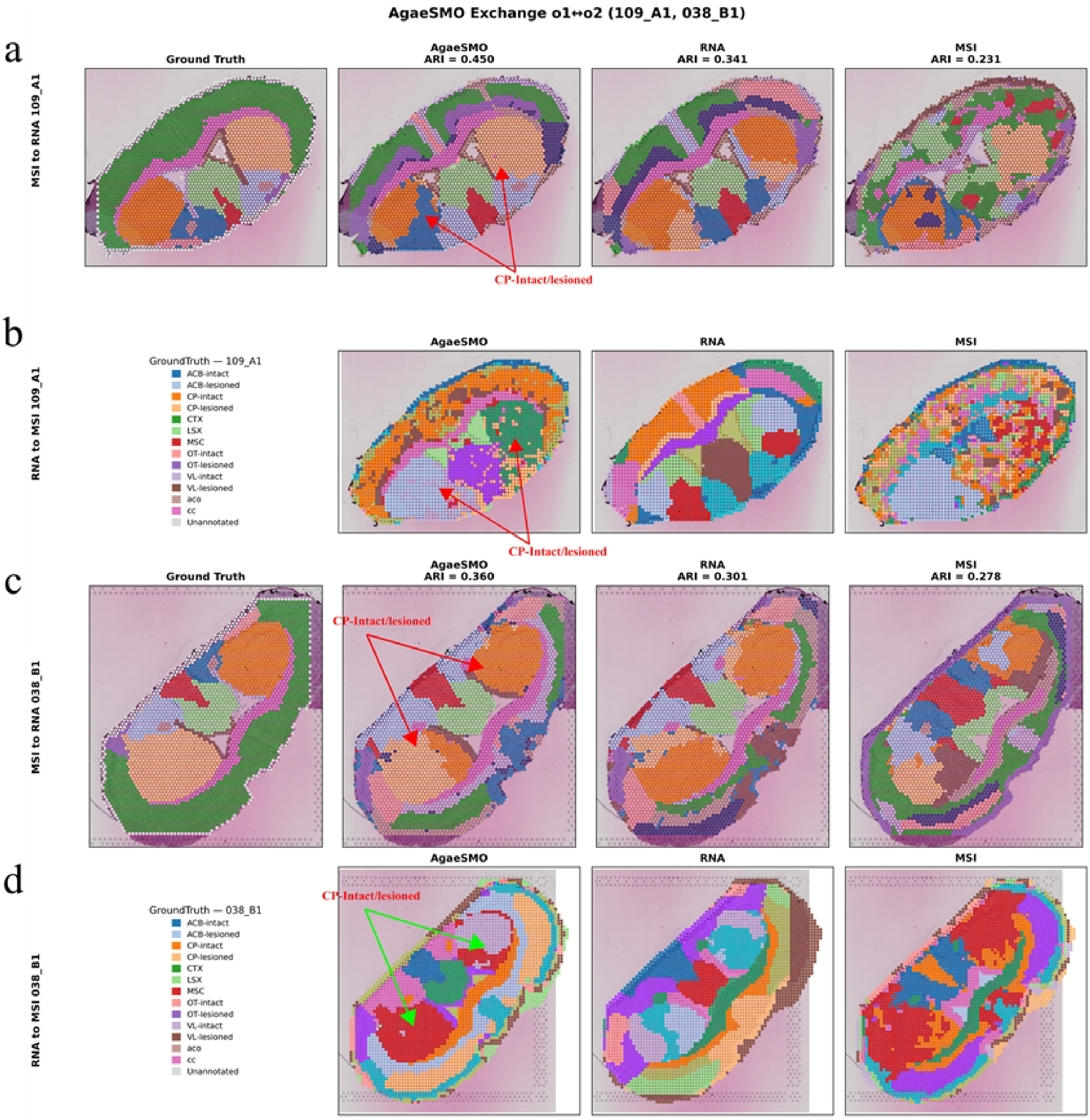

Supplementary Figure S12. Swapping study of RNA and MSI modalities. a Clustering results of aligning MSI to RNA using AgaeSMO on the109 A1 dataset. **b** Clustering results of aligning RNA to MSI using AgaeSMO on the 109 A1 dataset. **c** Clustering results of aligning MSI to RNA using AgaeSMO on the 038 B1 dataset. **d** Clustering results of aligning RNA to MSI using AgaeSMO on the 038 B1 dataset. CP-intact/ lesioned regions are indicated by arrows.

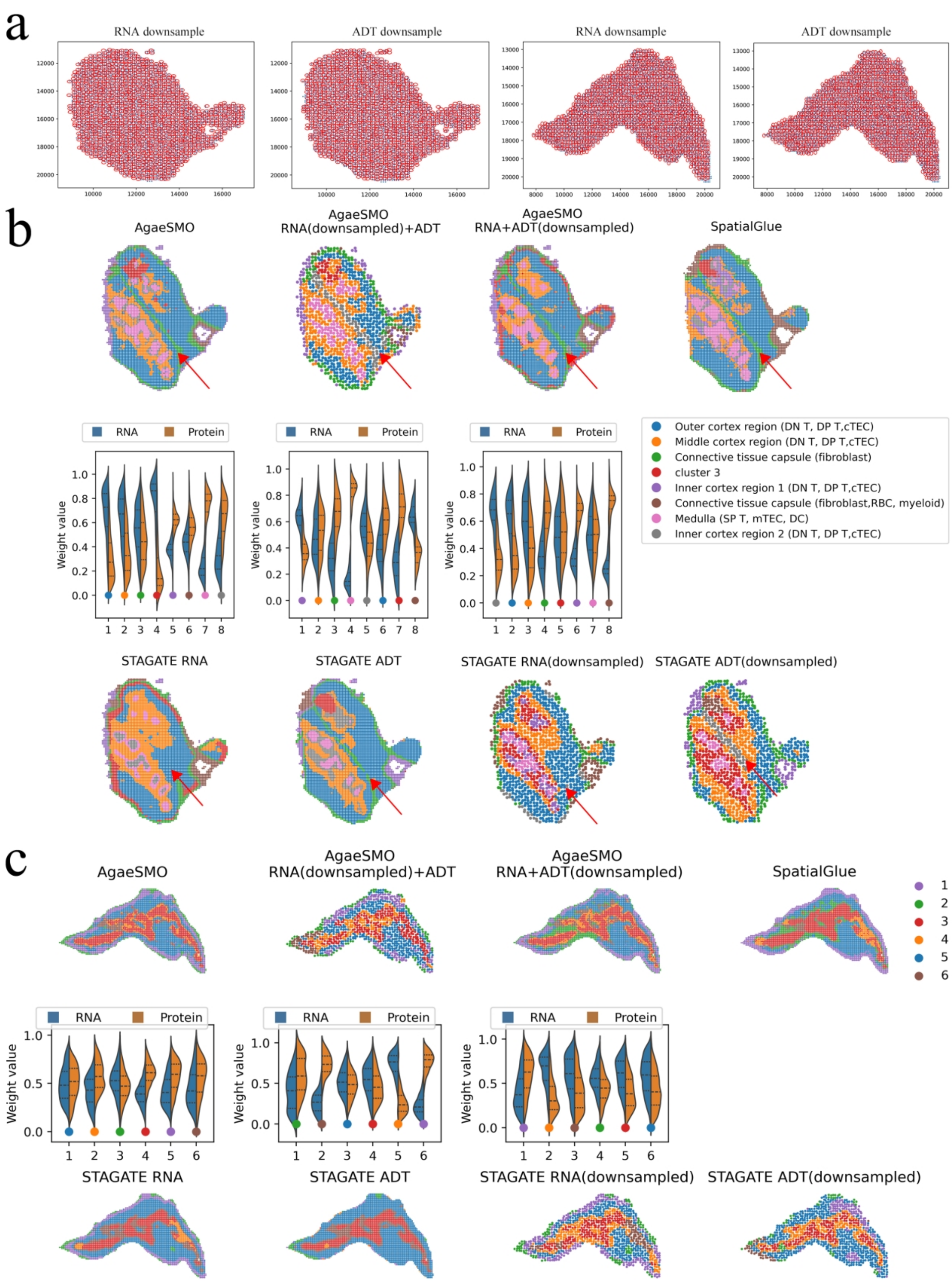

Supplementary Figure S13. Integration results of AgaeSMO on Mouse Thymus datasets with downsampled treatments comparing with STAGATE. a Downsampling method. Circle centers and radii were designated on the transcriptomic and proteomic data, respectively. The original spots within the radius were weighted averagely based on UMI counts. Random offsets were added to the coordinates of the center, with both x and y offsets drawn from a uniform distribution ranging from −50 to 50. **b** Clustering results of AgaeSMO on Mouse Thymus 1 for three combinations: (1) RNA+ADT, (2) RNA+ADT (downsampled), and (3) RNA (downsampled) +ADT, along with the corresponding modality weights, comparing with clustering results of SpatialGlue and STAGATE on the Mouse Thymus 1: full, RNA, ADT, RNA (downsampled), and ADT (downsampled) datasets. **c** Clustering results of AgaeSMO on Mouse Thymus 2 for three combinations: (1) RNA+ADT, (2) RNA+ADT (downsampled), and (3) RNA (downsampled)+ADT, along with the corresponding modality weights, comparing with clustering results of SpatialGlue and STAGATE on the Mouse Thymus 2: full, RNA, ADT, RNA (downsampled), and ADT (downsampled) datasets.

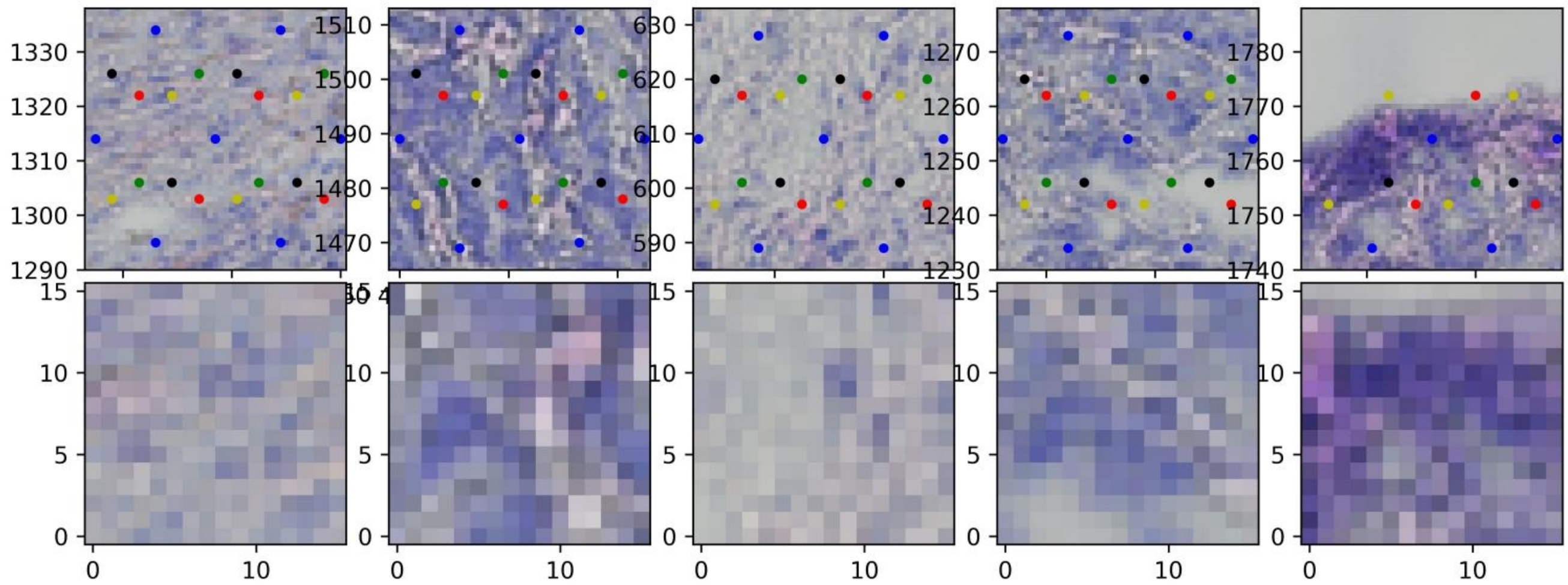

Supplementary Figure S14. Patches of H&E stained image of Human breast cancer datasets. Blue points represent centre of RNA-seq Spot, while red, yellow, black, green points represent anchored points centered around the blue dots. Interiors of these four points were flatten as H&E image embeddings corresponding to the blue dots.

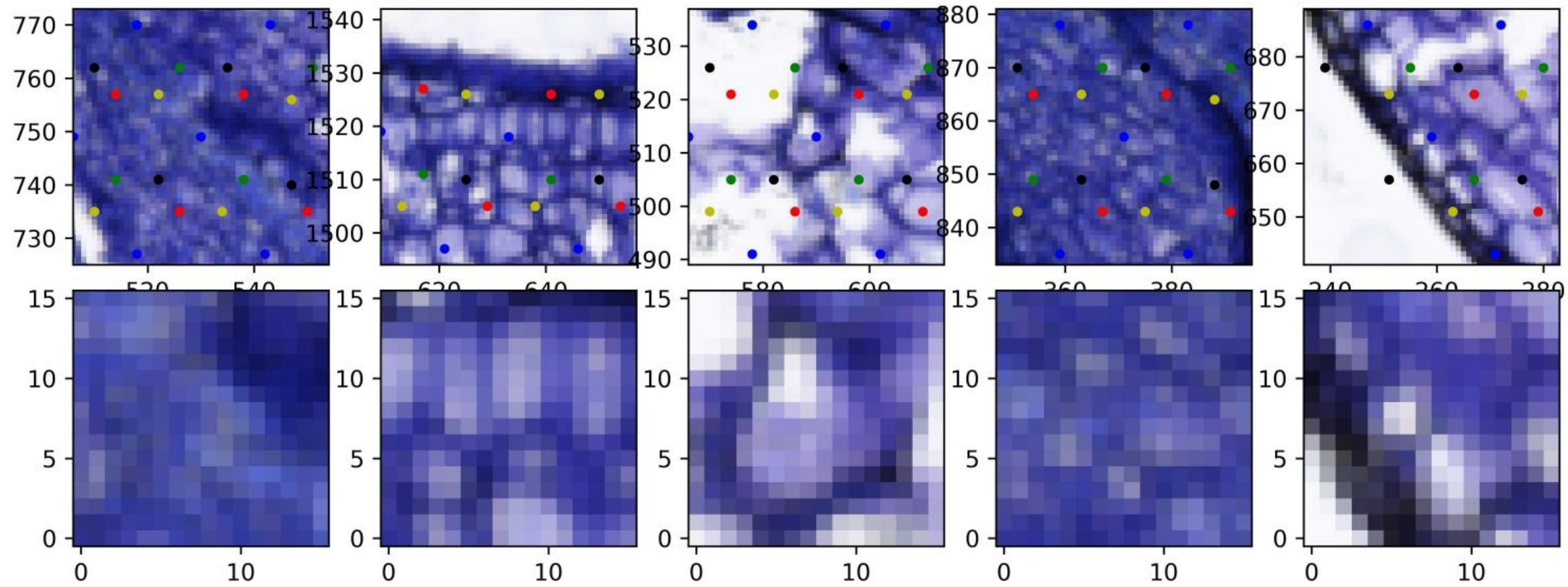

Supplementary Figure S15. Patches of H&E stained image patch of orchid *Phalaenopsis* flower bud datasets. Blue points represent center of RNA-seq spots, while red, yellow, black, green points represent anchored points centered around the blue dots. Interiors of these four points were flatten as H&E image embeddings corresponding to the blue dots.

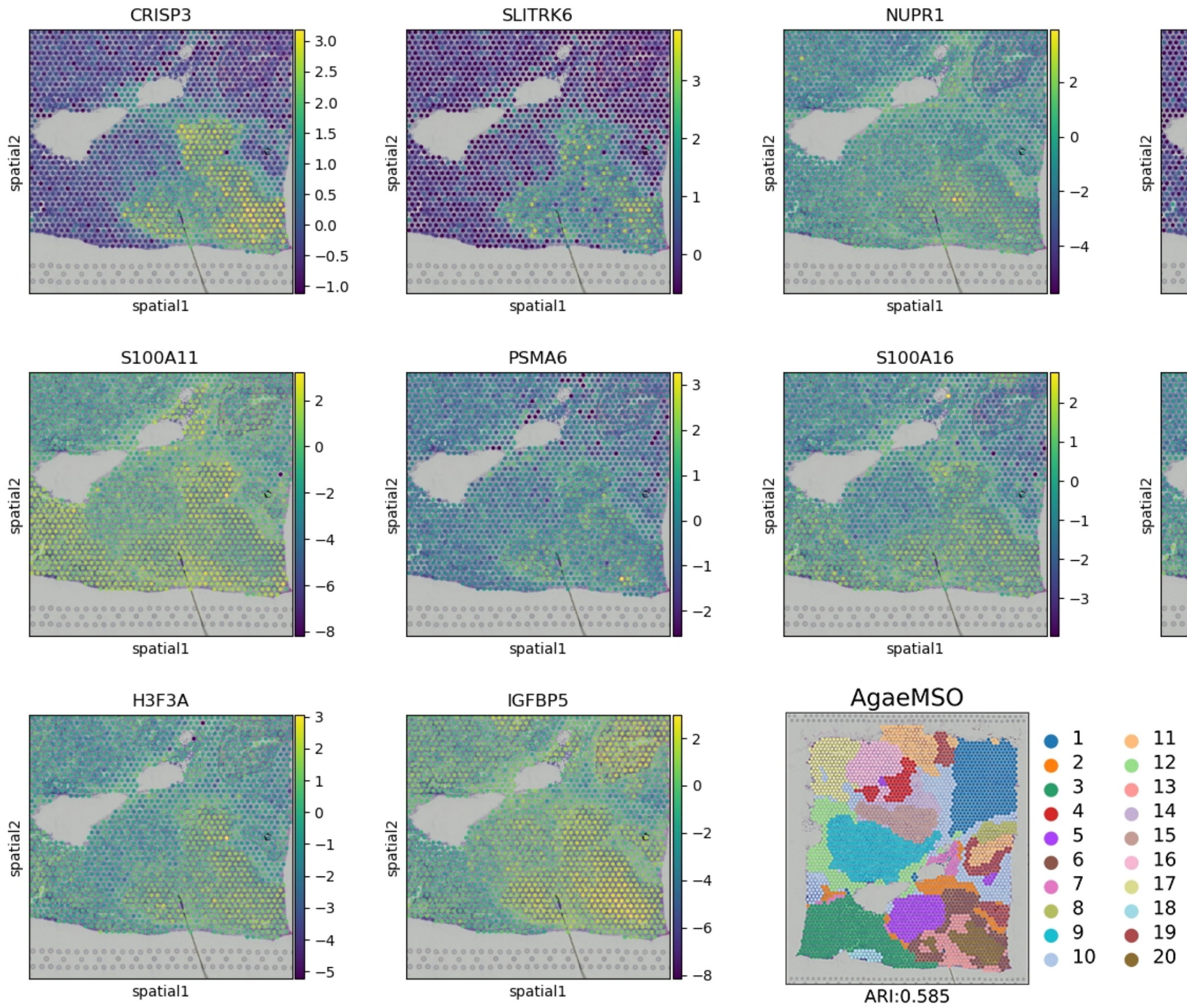

Supplementary Figure S16. In situ maps of top 10 DEGs between Group 13 versus rest regions in Human Breast Cancer dataset.

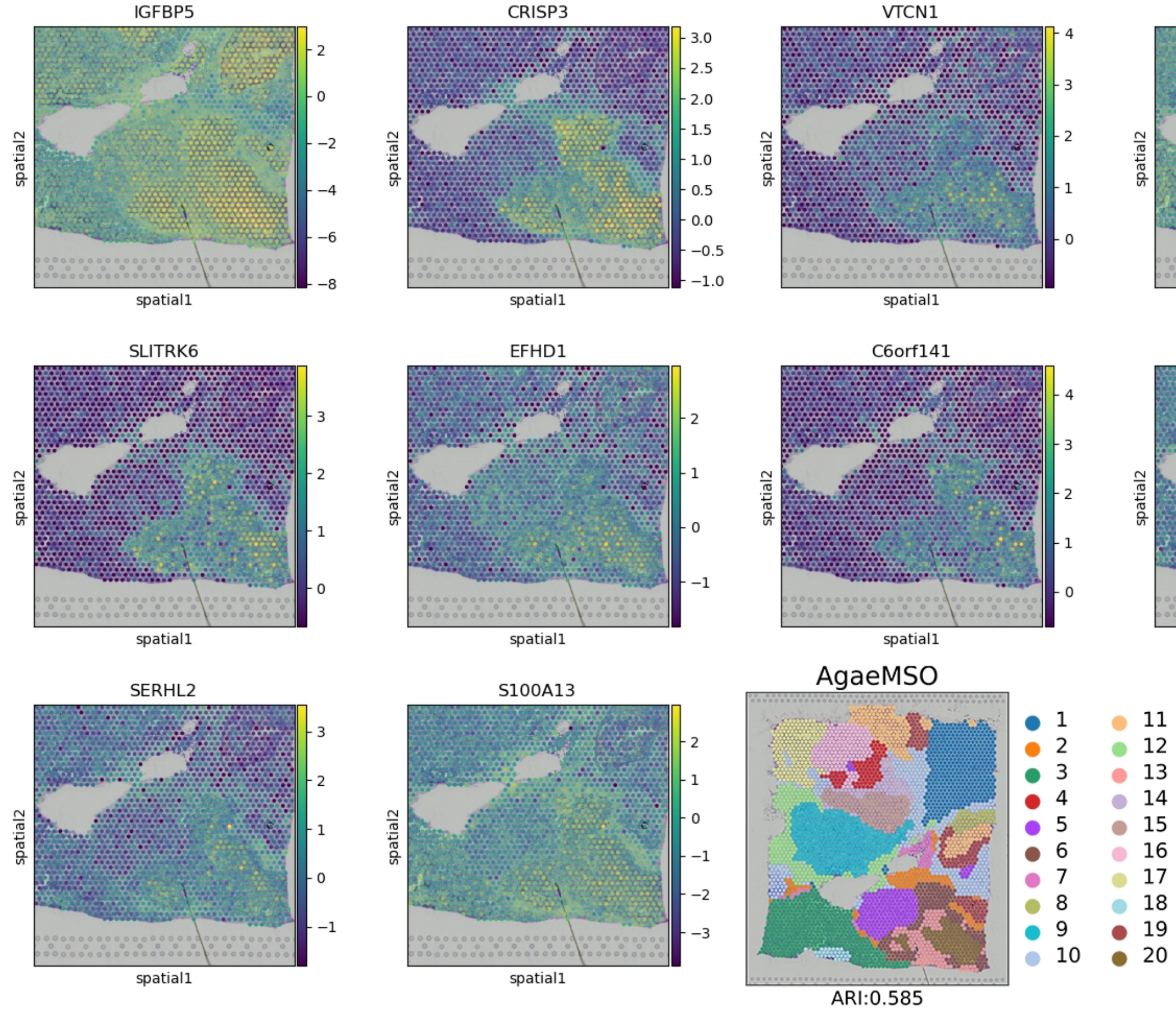

Supplementary Figure S17. In situ maps of top 10 DEGs between Group 20 versus rest regions in Human Breast Cancer dataset.

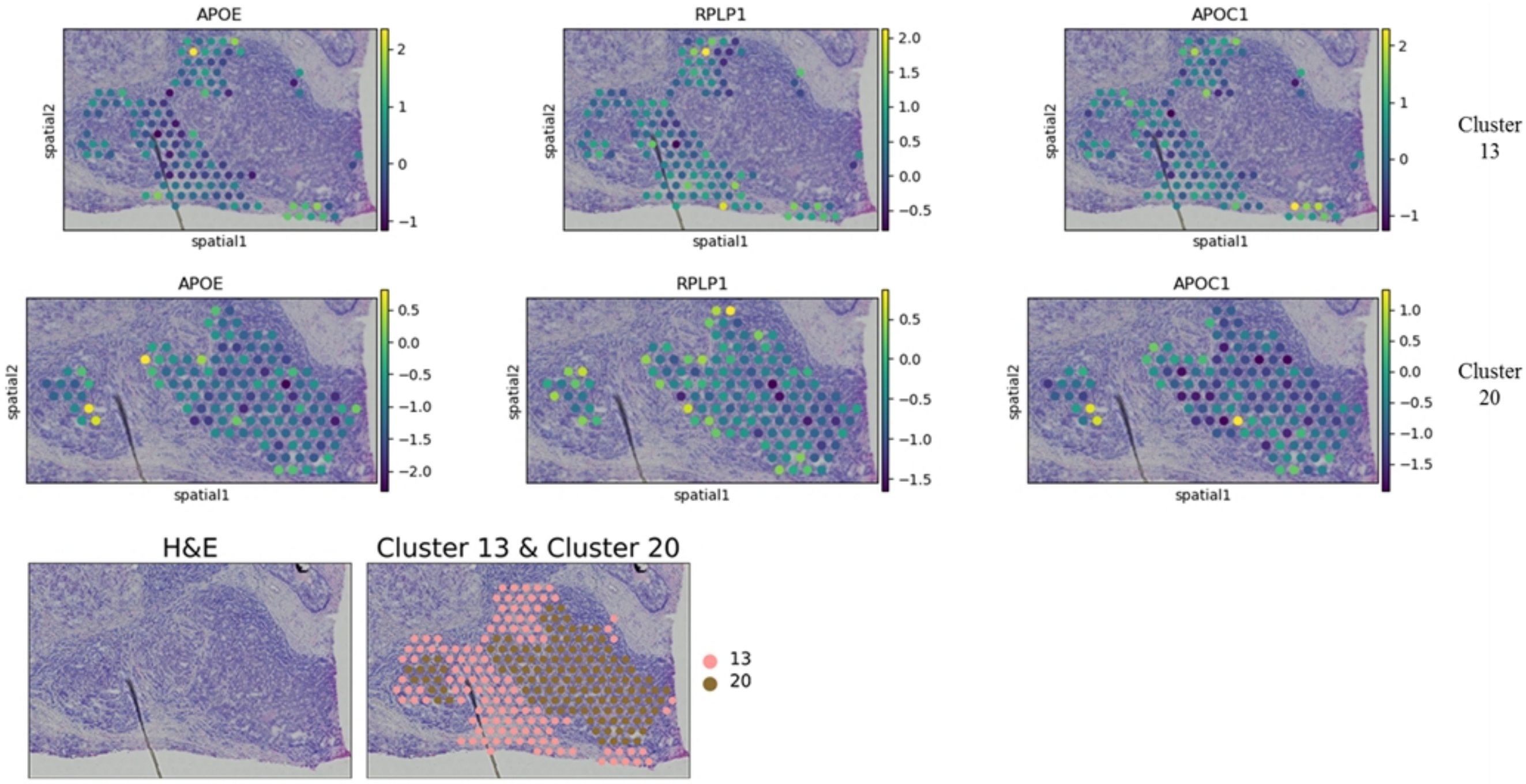

Supplementary Figure S18. In situ maps of top three DEGs between Group 20 versus Group 13 in Human Breast Cancer dataset.

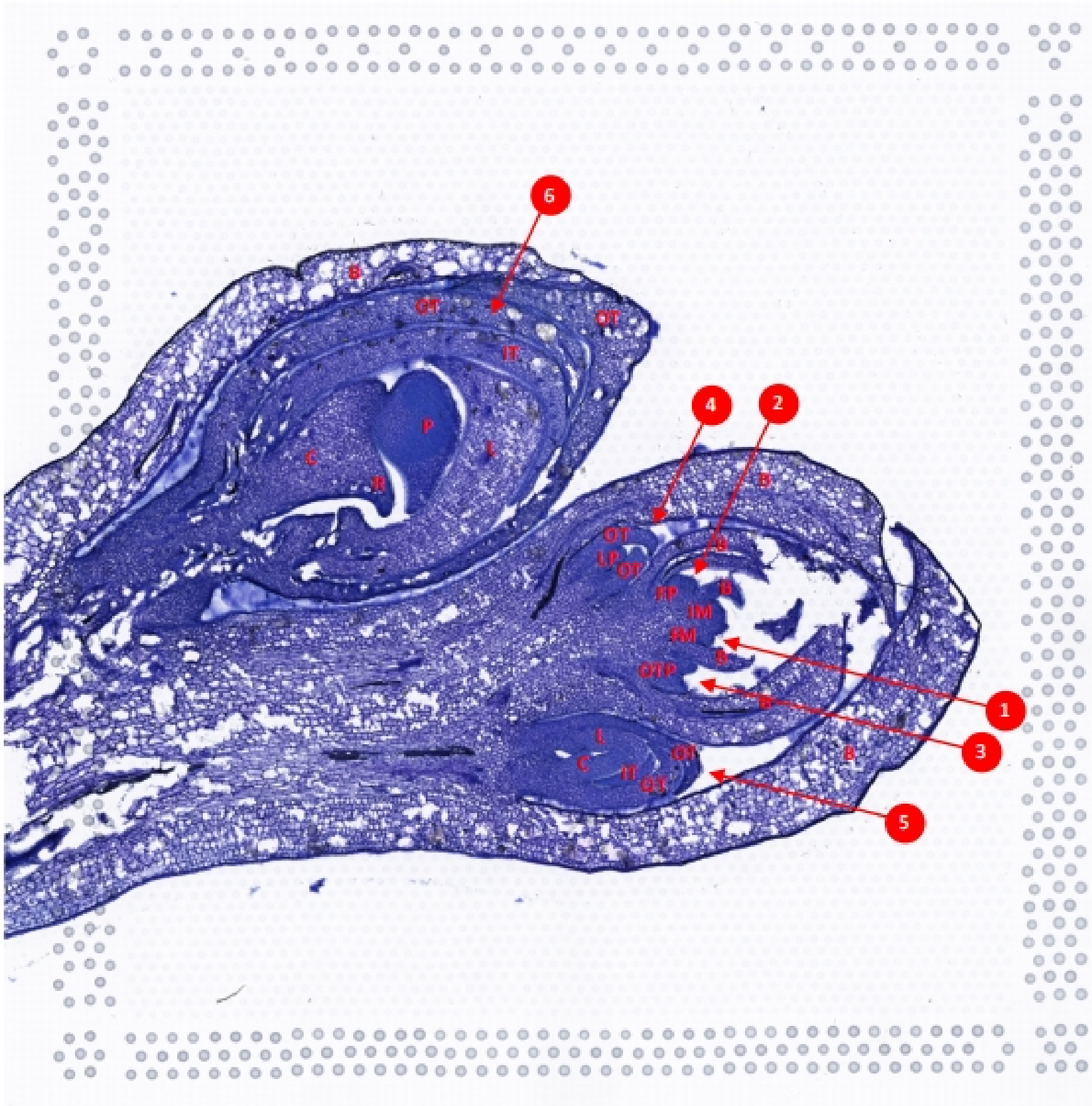

Abbr.	Tissue	Abbr.	Tissue	Abbr.	Tissue	Abbr.	Tissue
B	Bract	OT	Outer Tepal	C	Column	V	Viscidium
IM	Inflorescence Meristem	IT	Inner Tepal	CF	Column foot	T	Tegula
FM	Floral Meristem	L	Lip	P	Pollinium		
FP	Floral Primordium	LP	Lip Primordium	R	Rostellum		
OTP	Outer Tepal Primordium	LPJ	Lip Projection	AOA	Apex Of Anther		

Supplementary Figure S19. Manual annotation of orchid *Phalaenopsis* flower bud datasets.

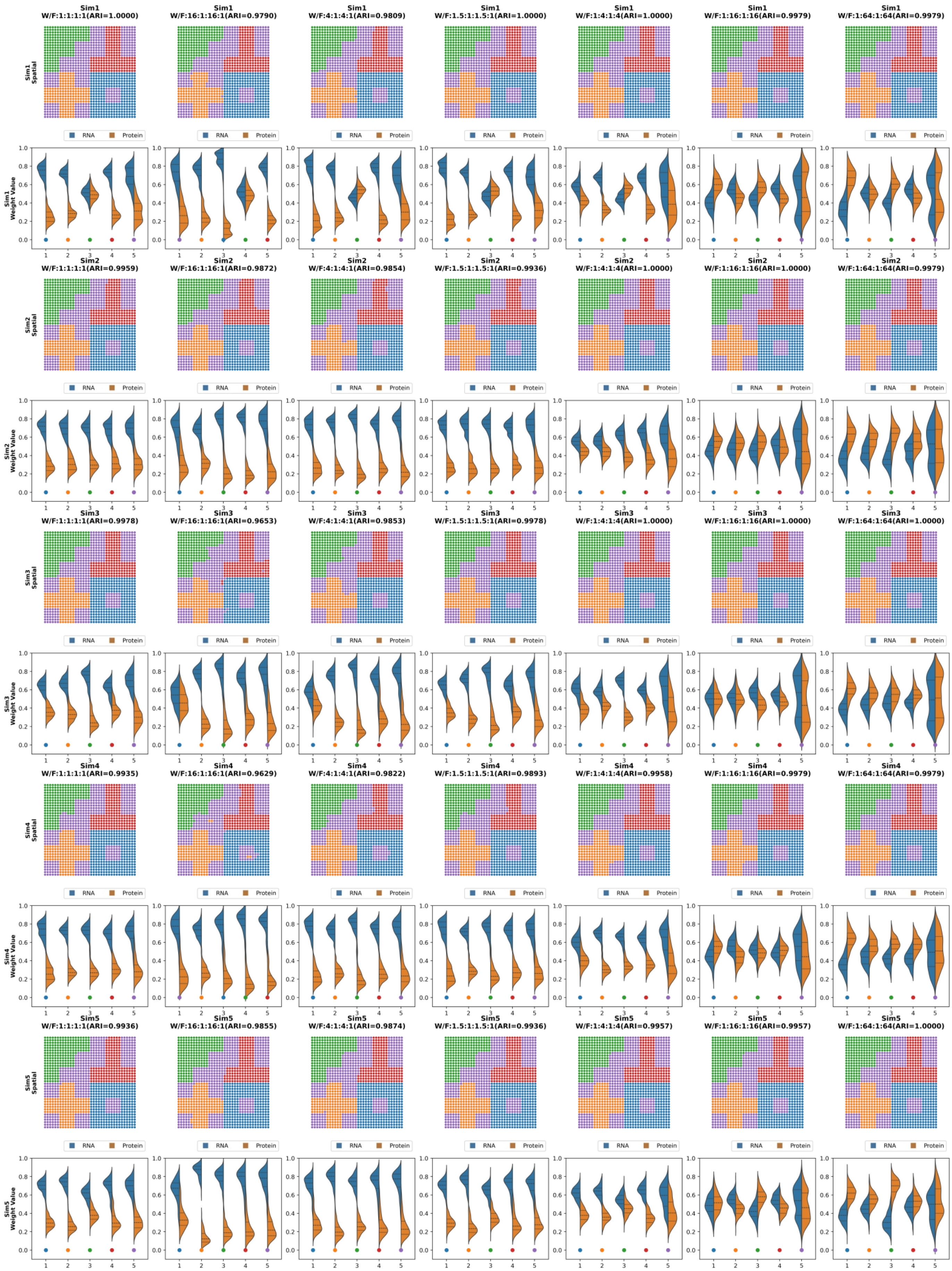

Supplementary Figure S20. Comparisons the modality weights (Violin Plot) of AgaeSMO under different weighting factors (W/F value) in five simulated datasets (sim1-sim5).

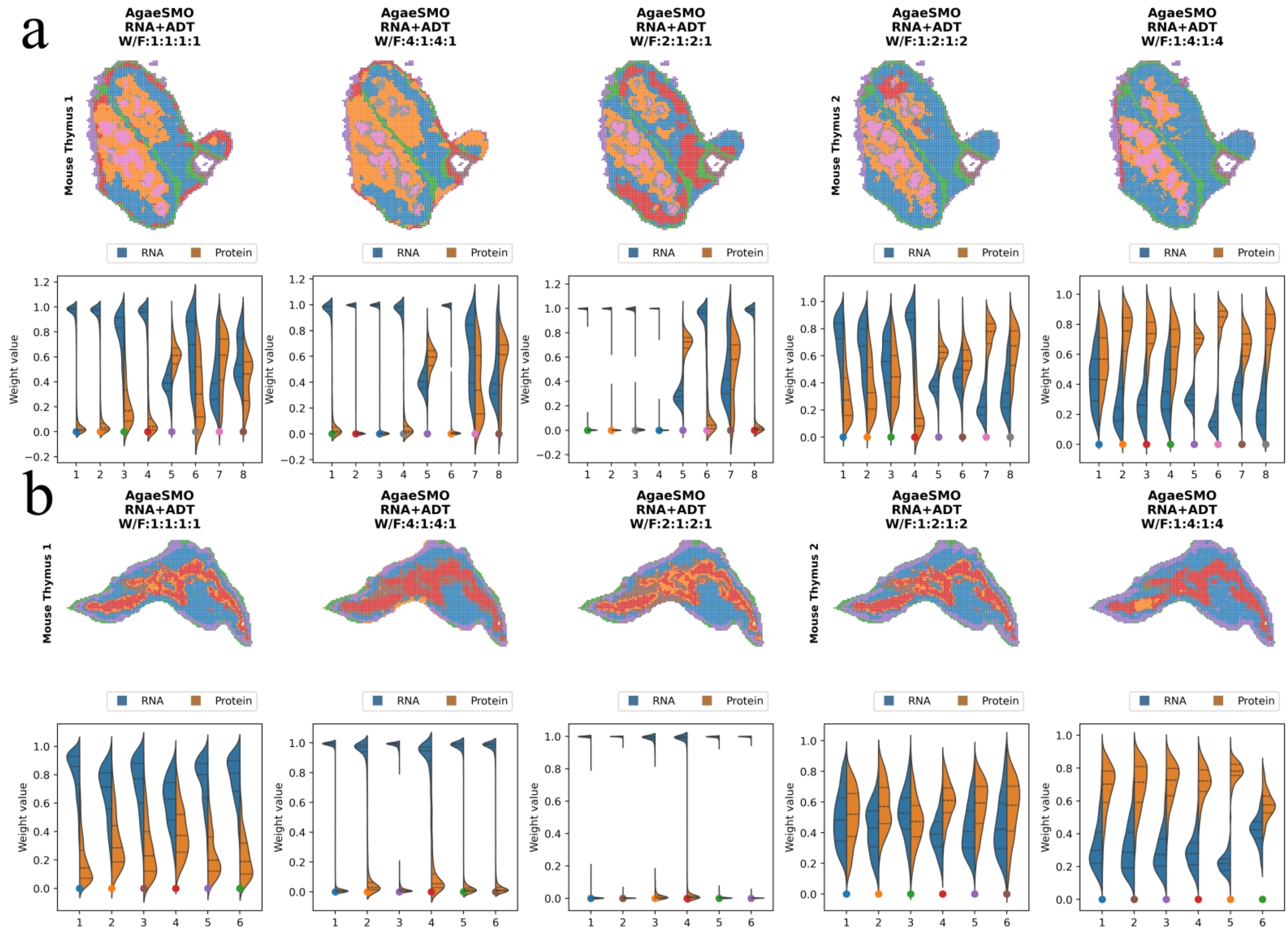

Supplementary Figure S21. Comparisons the modality weights (Violin Plot) of AgaeSMO under different weighting factors (W/F value) in two unpaired high-resolution spatial omics datasets. a In the mouse thymus 1 dataset, when W/F ratios = (4:1:4:1 or 2:1:2:1), the attention weights of most clusters are dominated by RNA modality. When W/F ratio = (1:2:1:2), the attention weights are no longer monopolized by single modality. At W/F ratio = (1:4:1:4), the attention weights are exclusively dominated by the Protein modality. **b** In the mouse thymus 2 dataset, when W/F ratios = (1:1:1:1; 4:1:4:1 or 2:1:2:1), the attention weights of all clusters are dominated by RNA modality. When W/F ratio = (1:2:1:2), the attention weights are no longer monopolized by single modality. At W/F ratio = (1:4:1:4), attention weights are exclusively dominated by the Protein modality.

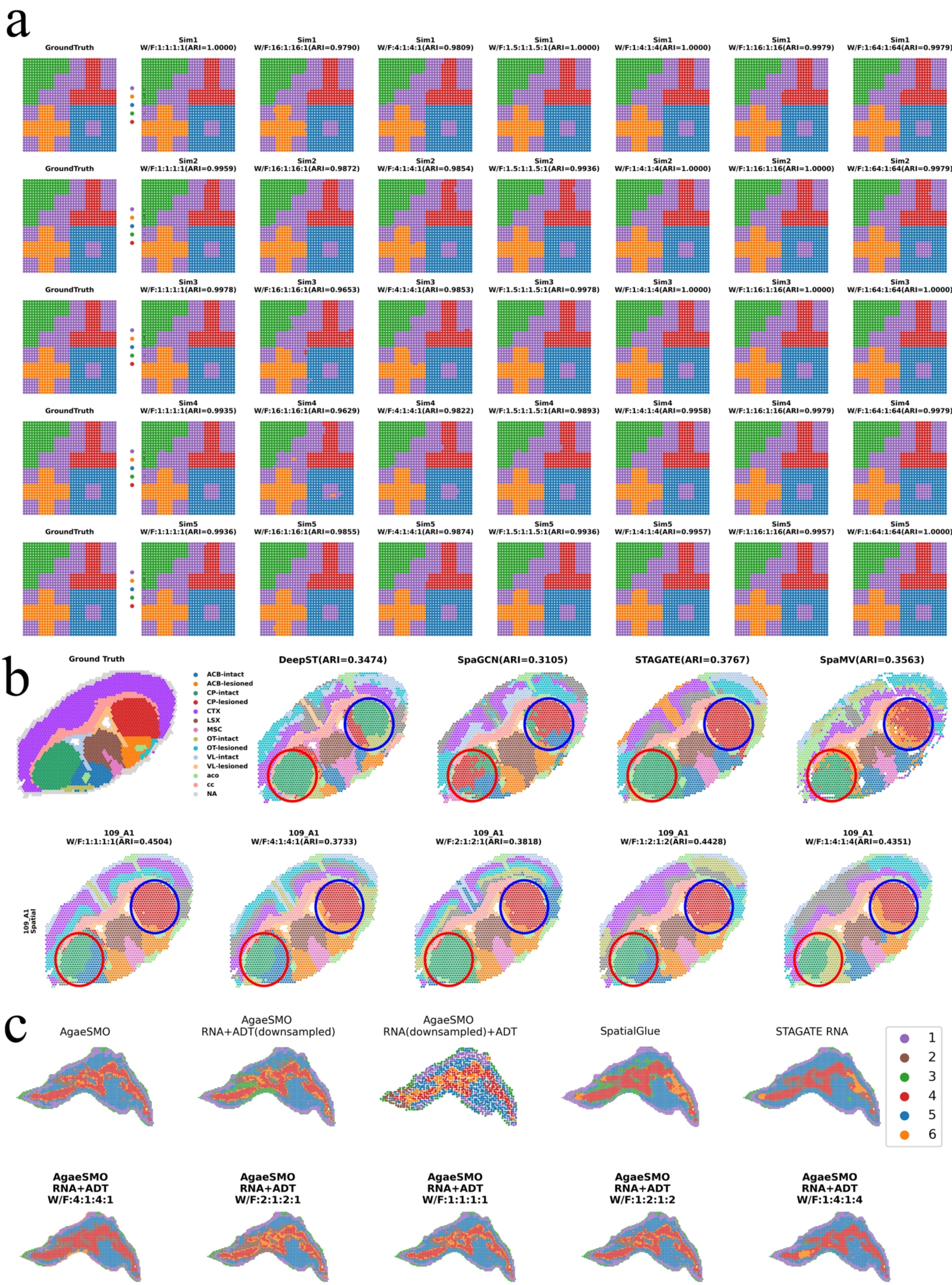

Supplementary Figure S22. The effects of weighing factor (W/F) on clustering performance across simulated and real datasets. a On the simulation dataset, when the W/F ratio was 1:1:1:1, all ARI values exceeded 0.99. Increasing the W/F ratio to 16:1:16:1 and 4:1:4:1 yielded ARI values ranging between 0.96 and 0.99 (for 16:1:16:1) and between 0.98 and 0.99 (for 4:1:4:1).

When the W/F ratio were reduced to 1.5:1:1.5:1, only Simulation Dataset 4 exhibited an ARI below 0.99. When the ratio scale differences of them became lager, the ARI values tended to stabilize, remaining above 0.99. **b** On the SMA 109 A1 dataset, after adjusting the W/F ratio, AgaeSMO still successfully distinguished between CP-intact and CP-lesioned regions. Only at W/F ratio of 4:1:4:1, the ARI fall slightly below that of STAGATE (with a difference of approximately 0.003). At all other W/F ratios, AgaeSMO outperformed the other methods. **c** On the mouse thymus 2 datasets, after adjusting the W/F ratio, AgaeSMO achieved clustering results largely comparable to those of SpatialGlue and STAGATE, with only minor differences in noise levels.
